# Nectar traits of New Zealand trees vary across climatic zones

**DOI:** 10.3389/fpls.2025.1539875

**Published:** 2025-10-03

**Authors:** Johanna M. van Delden, Sebastian Leuzinger, Sarah J. Richardson, Michael J. Clearwater

**Affiliations:** ^1^ Ecology, Biodiversity and Animal Behaviour, Te Aka Mātuatua – School of Science, University of Waikato, Hamilton, New Zealand; ^2^ School of Science, Auckland University of Technology, Auckland, New Zealand; ^3^ Manaaki Whenua – Landcare Research, Lincoln, New Zealand

**Keywords:** flower traits, regional variation, Pittosporum, Sophora, Fuchsia, Metrosideros, Leptospermum, Cordyline

## Abstract

**Introduction:**

To evaluate whether plant traits (nectar volume, concentration, sugar mass, flower fresh mass, and size) vary regionally in response to climate, we examined eight native New Zealand tree species.

**Methods:**

Flowers were sampled using micropipettes from seven sites across five climate zones spanning both main islands (37–45°S/170–177°E) after having been bagged for 24 hours. Trait data were standardized (0–1 scale) and pooled into a global dataset for cross-species analysis. We used linear regression to assess correlations between plant traits across and within species, followed by parametric and non-parametric tests to examine regional variation. Generalized additive mixed models (GAMMs) were applied to model trait responses to regional climate factors, identifying significant correlations within and across species.

**Results:**

Sampling yielded 4,276 flowers and 2,240 μL of nectar from 164 trees. Nectar volume ranged from 0.3–72 μL, concentration from 0.4–53°Brix, sugar mass from 0.01–13 mg, flower fresh mass from 4–1116 mg, and flower size from 4–54 mm. Across species, nectar concentrations were generally higher in drier regions (Canterbury and Hawke’s Bay) located in the rain shadow of axial mountain ranges on New Zealand’s east coast. Nectar volumes and flower masses were greatest in Dunedin, likely influenced by high relative humidity and low sunshine hours. In Nelson-Tasman and Marlborough, flowers were larger, but this trend was unexplained by climatic factors. Within species, plant traits exhibited regional variation, with highly species-specific trait relationships. GAMMs revealed significant climate-trait correlations in 87.5% of species, with climate variables explaining 18–84% of regional variation. Annual sunshine hours and rainfall had the strongest effects, and South Island nectar contained the highest sugar amounts in 67% of species.

**Discussion:**

Although no uniform trend was evident across species, nectar volumes tended to be lower in sunnier regions, while flowers were larger and nectar concentrations higher in drier areas. Future studies should examine closely related species with larger sample sizes per region, ideally incorporating microclimate data from standardized measurement periods prior to sampling.

## 1 Introduction

Abiotic regional differences exert various pressures on local plant populations, leading to genotypic and phenotypic variation, such as changes in flower size (e.g. Domínguez et al., 1998; Hattori et al., 2015; Garcia et al., 2021). Interspecific variability in nectar volume and composition among plant species is well-documented (e.g. Percival, 1961; Baker and Baker, 1983; Palmer-Young et al., 2018), but intraspecific variation in nectar characteristics remains less well-understood (Lanza et al., 1995).

Other than being influenced by biotic factors such as microbial colonization and animal pollinator visits (e.g. Herrera et al., 2008; Keasar et al., 2008; Mittelbach et al., 2015), nectar characteristics may vary within species due to environmental factors such as solar radiation (e.g. Shuel, 1952; Pleasants, 1983; Boose, 1997), ambient humidity/vapor pressure (e.g. Shuel, 1952; Chabert et al., 2020), air temperature (e.g. Shuel, 1952; Clearwater et al., 2018; Noe et al., 2019), rainfall/plant water status (e.g. Wyatt et al., 1992; Keasar et al., 2008; Clearwater et al., 2018), and soil nutrient concentrations (e.g. Nickless et al., 2017).

Solar radiation has a strong and consistent influence on nectar production and sugar secretion across multiple plant species. In controlled glasshouse experiments with *Trifolium pratense* L. (red clover, Fabaceae), increased solar radiation was found to be highly and directly correlated with nectar sugar secretion (Shuel, 1952). Nectar sugar production in *Ipomopsis aggregata* (Pursh) V.E. Grant (scarlet gilia, Polemoniaceae) was shown to decrease by 47% over 24 hours on overcast days compared to sunny days, reinforcing earlier findings (Pleasants, 1983). In greenhouse-grown *Epilobium canum* (Greene) P.H. Raven (California fuchsia, Onagraceae), nectar production rates were significantly influenced by light availability, with plants grown under a 70% reduction in ambient light producing 27% less nectar than controls (Boose, 1997). Solar radiation also accounted for significant between-site (n = 5) variation in nectar sugar amount and composition in wild-grown *Leptospermum scoparium* L. (mānuka, Myrtaceae) in New Zealand (Noe et al., 2019).

Atmospheric moisture conditions, such as vapor pressure and vapor pressure deficit (VPD), can influence nectar concentration and volume, though effects vary by species. Higher vapor pressure during the 12 hours prior to nectar extraction was found to be strongly inversely correlated with nectar concentration in red clover, accompanied by a highly significant, though weaker, direct correlation with nectar volume (Shuel, 1952). In contrast, a positive relationship between nectar concentration and vapor pressure deficit was reported when nectar secretion was studied in 34 sunflower (*Helianthus annuus* L., Asteraceae) hybrids under controlled environmental conditions (Chabert et al., 2020).

Air temperature can influence nectar traits, though its effects may depend on timing, species, and interaction with other environmental factors. Investigations into the effects of air temperature on nectar production in red clover revealed no significant differences between plants exposed to a mean night temperature of 60°F and those at 70°F under similar daytime conditions (Shuel, 1952). In contrast, night-time temperature was found to contribute partially to intraspecific nectar variation in *Leptospermum scoparium*, with air temperature and solar radiation together explaining 80% of site variation (Noe et al., 2019). Nectar sugar amounts in the same species were also positively correlated with the daytime temperature of the preceding day rather than the day of collection (Clearwater et al., 2018).

Water availability, rainfall, and soil conditions significantly influence nectar production, often in interaction with plant genotype and species-specific responses. Nectar production rates in *Epilobium canum* were also found to be significantly influenced by water availability, with plants grown under reduced watering over a 14-week period—receiving only 25% of the water given to controls—producing 26% less nectar (Boose, 1997). Similarly, drought stress reduced sugar production in *Silphium perfoliatum* L. (cup plant, Asteraceae) (Mueller et al., 2020), and both nectar volume and total sugar amounts in *Epilobium angustifolium* L. (fireweed, Onagraceae), although concentration remained unchanged (Carroll et al., 2001). In contrast, some species such as *Ipomopsis longiflora* (Torr.) V.E.Grant (flaxflowered gilia, Polemoniaceae) (Villarreal and Freeman, 1990) and *Leptospermum scoparium* (Clearwater et al., 2018) showed little or no response to lower water availability. Other studies have reported positive effects of rainfall on nectar volumes the day prior to sampling (Wyatt et al., 1992; Keasar et al., 2008). In one such experiment, simulated rainfall equivalent to 10 cm increased nectar volume and sucrose amounts approximately twofold in *Asclepias syriaca* L. (common milkweed, Apocynaceae) (Wyatt et al., 1992). Variation in nectar volumes also appears to have a heritable component, as shown in *Echium vulgare* L. (viper's bugloss, Boraginaceae) genotypes, although water availability significantly influenced nectar output as well (Leiss and Klinkhamer, 2005). Increased water availability particularly boosted nectar production in genetically low-producing lines, highlighting a genotype-by-environment interaction. High-nectar lines exhibited greater root mass and drought resilience, maintaining higher nectar output under dry conditions. Field observations further indicated that additional watering increased nectar production across all genetic lines. In New Zealand, regional nectar variation in *Leptospermum scoparium* has been attributed to both environmental and phylogenetic influences (Williams, 2012), a conclusion supported by subsequent findings (Noe et al., 2019). Additionally, soil type variation was shown to interact with genotype to influence nectar yield in three *Leptospermum scoparium* cultivars, despite soil type alone having no significant effect on nectar composition or production (Nickless et al., 2017).

Nectar research in New Zealand has often focused on the contribution of nectar to pollinator energetics, incorporating a limited number of plant species and sites, or relying on estimated rather than measured nectar values (Whitaker, 1987; Rasch and Craig, 1988; Butz Huryn, 1995; Castro and Robertson, 1997; Ladley et al., 1997; Murphy and Kelly, 2003; Ausseil et al., 2018), with the exception on nectar variation in *Leptospermum scoparium*, based on the species being highly valuable for the honey industry (e.g. Nickless et al., 2017; Clearwater et al., 2018; Noe et al., 2019).

One reason for the lack of information on regional variation in nectar production by individual species is the difficulty of collecting nectar samples. Accurately sampling nectar, given its dynamic nature and the multitude of influencing floral factors, presents significant challenges for research (e.g. Petit et al., 2011; Faegri and van der Pijl, 2013; Brito Vera and Pérez, 2024).

Given these challenges, our study aims to examine the influence of various abiotic factors on the nectar traits of native New Zealand tree species, utilizing the country’s diverse climatic zones, which range from warm temperate in the north to cooler temperate in the south, influenced by a broad latitudinal range and complex geography (Kidson, 2000; NIWA, 2018). This unique setting allows us to explore nectar trait variability across different environmental conditions.

Building on previous studies that show the impact of environmental factors on nectar traits, we questioned the extent to which common New Zealand species vary in their nectar-related responses across different climatic regions. Understanding this variation could help scale nectar production across landscapes. It also raises important questions about the reliability of using species averages to predict nectar abundance and composition and whether environmental variables should be included in models predicting nectar availability across landscapes.

Our study aims to clarify the influence of climate on nectar (volume, sugar, concentration) and floral (flower fresh mass and size) traits (grouped as ‘plant traits’) variation in common tree species across diverse coastal climate zones in New Zealand. We hypothesized that relationships between (1) climate drivers and plant traits, and (2) floral and nectar traits would be consistent across species. For climate effects, we expected that (a) nectar volumes would be highest in regions with high humidity due to nectar’s hydrophilic nature; (b) nectar concentrations would be highest in the driest regions, particularly on the east coasts of both islands, where limited precipitation and higher evaporation rates are common; and (c) nectar sugar mass, flower fresh mass, and flower size would be highest in regions with the most sunshine hours, which support photosynthesis and the growth of larger, sugar-rich flowers.

## 2 Materials and methods

### 2.1 Study sites

**Figure 1:**
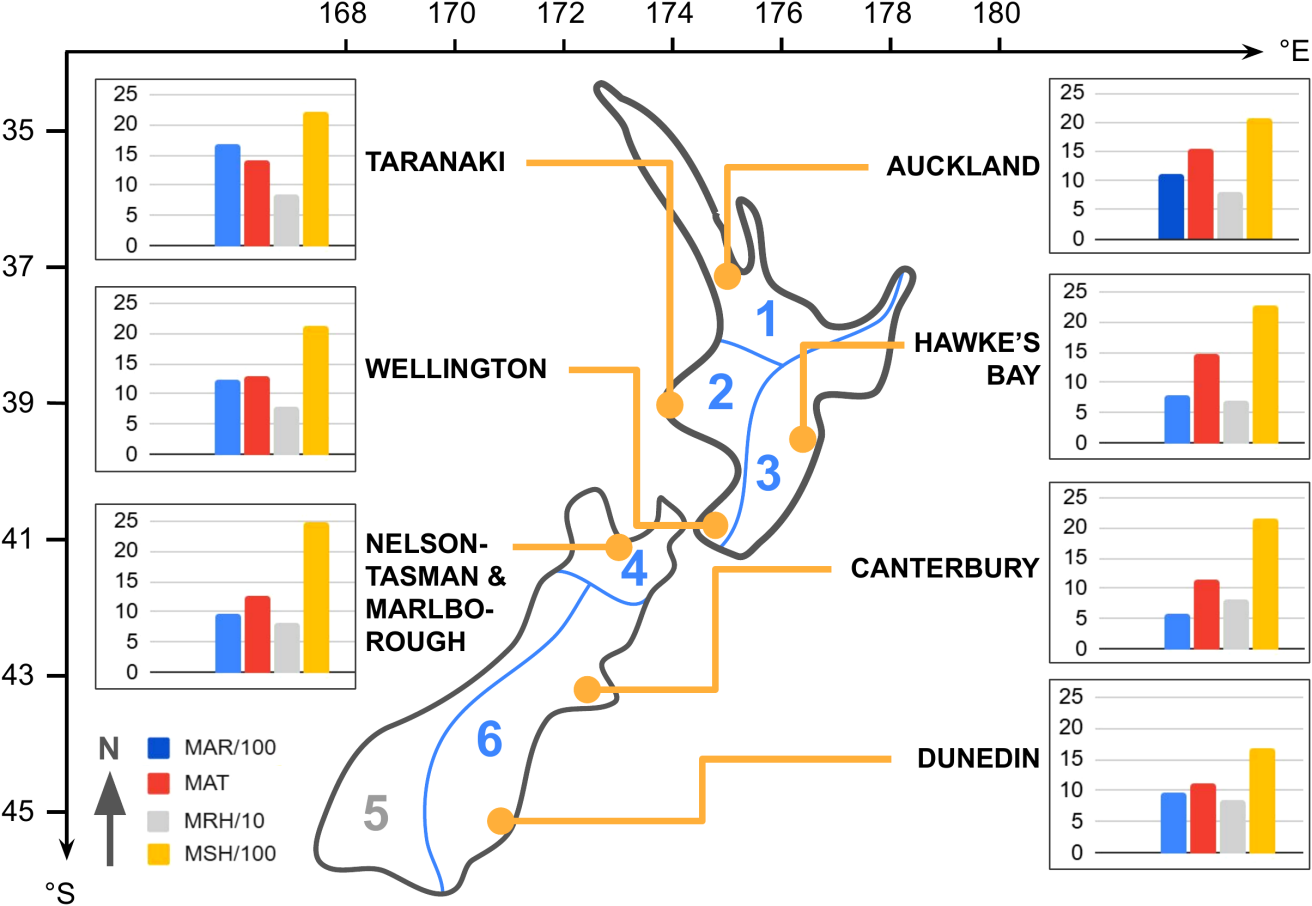
Geographical locations and regional climate data of the seven sampling sites in New Zealand’s six climate zones (orange dots; climate zones labeled in blue, simplified zoning boundaries in alignment with suggested areas by Kidson, 2000). Latitude (°S) and longitude (°E) coordinates are provided. Regional mean annual climate data is displayed for each site, with transformed scales for consistency: blue bars (MAR/100) represent mean annual rainfall (mm*100), red bars (MAT) indicate mean annual temperature (°C), gray bars (MRH/10) show mean annual relative humidity (%*10), and yellow bars (MSH/100) denote mean annual sunshine hours (h*100).

Sampling was conducted across eight New Zealand sites between 37–45°S and 170–177°E (**Table 1** & **Figure 1**). On New Zealand’s North Island, we sampled Auckland (north), Taranaki (west), Hawke’s Bay (east), and Wellington (south). On the South Island, we collected flowers from Nelson-Tasman/Marlborough (north), West Coast (west), Canterbury (east), and Dunedin (south). Due to insufficient sample sizes, we excluded the West Coast site from further analysis.

**Table 1:** Overview of sample sites (with all species sampled across all sites; n _trees/sp._ ~ 3), climatic 30-year norms (NIWA, 2018), and sampling details, with ‘MAR’, mean annual rainfall in mm/a; ‘MAT’, mean annual air temperature in °C; ‘MRH’, mean annual relative humidity in %; ‘MSH’, mean annual sunshine hours; ‘LAT’, latitude in °S; ‘LON’, longitude in °E; ‘Flowers emptied’, number of flowers sampled for nectar; ‘Flowers measured’, number of flowers measured for size; ‘Flowers weighed’, number of flowers weighed for fresh mass).

Sample sites and climate								Sampling amounts				
Site	Climate zone	MAR	MAT	MRH	MSH	LAT	LON	Trees	Flowers			Nectar
		mm	°C	%	h	°S	°E	n	Emptied	Measured	Weighed	µL
Auckland	Northern North Island	1119	15.6	81	2062	36.8	174.7	22	395	398	366	5000
Taranaki	South-West North Island	1683	14	85	2197	39	174	21	466	382	618	6147
Wellington		1249	12.8	79	2110	41.2	174.7	24	410	414	560	5132
Hawke’s Bay	Eastern North Island	786	14.6	71	2265	39.5	176.8	23	432	423	551	5877
Nelson-Tasman/Marlborough	Northern South Island	959	12.7	82	2472	41.2	173.2	26	471	483	563	6373
Canterbury	Eastern South Island	594	11.6	81	2143	43.5	172.6	24	428	410	628	4578
Dunedin		968	11.1	85	1681	45.8	170.5	24	620	489	990	11334

Because each site was sampled multiple times over two flowering seasons (2019/20) based on each species’ phenology—which peaked at different times along a north-to-south gradient (37°S to 45°S, spanning approximately 1,500 km)—we opted to use annual mean climate data in our analysis to examine plant trait variation in relation to general climate rather than daily weather. This approach simplified the identification of broad trends, avoiding the considerable complexity of analyzing daily microclimate data from 164 samples across seven sites and eight species. Although this method may mask some underlying effects, it still provides a resolution of results suitable for discussing overall trends.

In the seven analyzed sites, the annual mean air temperature (MAT) ranged from 11.1–15.6 °C, the annual mean relative humidity (MRH) from 71–85%, the annual sunshine hours (MSH) from 1681–2472 hours, and the annual mean rainfall (MAR) from 594–1683 mm (based on 30-year norms, 1981–2010, for each sample region, NIWA, 2018). The sample sites fell within five of the six climate zones identified by Kidson (2000) (**Table 1** & **Figure 1**).

### 2.2 Species

**Figure 2:**
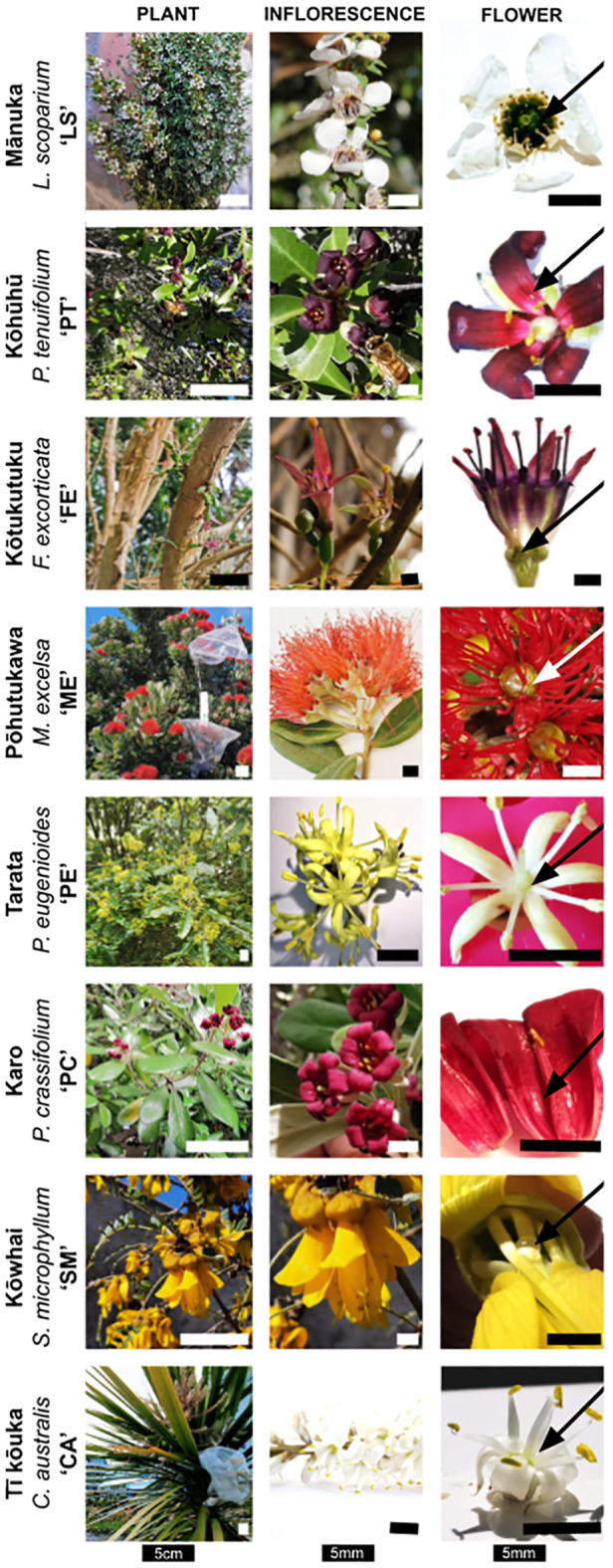
Photos of the eight sampled species (rows), with columns showing the overall appearance, whole flower or inflorescence, and a detailed close-up of its nectar secretion with arrows indicating the location of nectar within the flower (photographs: JD) and bars indicating the shown structure’s size. The scale bars in the left column (‘Plant’) represent 5 cm, while all other scale bars indicate 5 mm.

Based on their broad distribution across New Zealand (Martin, 1961), this study analyzed eight species (**Figure 2**). The selected species included three Asterids: karo (*Pittosporum crassifolium* Banks & Sol.; henceforth abbreviated as ‘PC’ in figures and tables, Pittosporaceae), kōhūhū (*Pittosporum tenuifolium* Gaertn.; ‘PT’), and tarata (*Pittosporum eugenioides* A. Cunn.; ‘PE’). Additionally, we sampled four Rosids, including three members of the Myrtales: kōtukutuku (*Fuchsia excorticata* L. f.; ‘FE’, Onagraceae), pōhutukawa (*Metrosideros excelsa* A. Cunn. ex G. Don; ‘ME’, Myrtaceae), and mānuka (*Leptospermum scoparium* J. R. Forst. & G. Forst.; ‘LS’, Myrtaceae), and one member of the Fabales: kōwhai (*Sophora microphylla* Aiton; ‘SM’, Fabaceae). Lastly, we analyzed tī kōuka (*Cordyline australis* Forster; ‘CA’, Asparagaceae), representing monocotyledons.

### 2.3 Sampling

We sampled all eight species across seven sites with an average of three trees per species at each site, totaling 164 trees (**Tables 1**–**3**, **Supplementary Tables S1a–h**). Across all sample sites, we collected flowers from 18 trees each from tī kōuka and kōhūhū, 19 trees from tarata, 20 trees each from mānuka and pōhutukawa, 21 trees each from karo and kōwhai, and 27 trees from kōtukutuku. The sampled individuals were predominantly naturally occurring wild plants growing in parks and forest reserves. Some individuals may have been planted, but ornamental cultivars were excluded. During each species’ peak anthesis period (iNaturalist, 2018–20; Newstrom-Lloyd, 2013) in the spring and summer of 2019 and 2020, we selected 20–40 fully opened flowers from each tree, totaling 340–889 flowers per species and 4,276 flowers overall.

**Table 2:** Overview of species–specific sampling ranges (top row) and means (± standard deviation; bottom row) across individuals.

Species		Nectar				Flowers						
Name	Trees	Samples	Sugar/ Flower	Concentration	Volume/ Flower	Fresh mass	Size	Per nectar sample	Nectar removed from	Weighted/ Tree		Measured/ Tree
	n	n	(mg)	(Brix)	(µL)	(mg)	(mm)	n	n	n	sum	n
*Cordyline australis*	18	18	0.03–0.44	1–12	2–4	8–34	5–9	15–20	345	20–50	440	16–33
			0.2 ± 0.1	6 ± 3	3 ± 1	19 ± 6	7 ± 1	19 ± 2		24 ± 10		20 ± 3
*Fuchsia excorticata*	27	27	0.04–7.11	2–25	2–47	68–512	10–28	5–44	492	6–50	633	11–38
			3.3 ± 2.4	13 ± 6	23 ± 13	240 ± 91	18 ± 5	18 ± 10		23 ± 13		19 ± 6
*Leptospermum scoparium*	20	20	0.01–0.6	1–15	0–9	13–86	9–17	20–40	460	19–40	519	18–40
			0.2 ± 0.2	6 ± 4	3 ± 2	40 ± 15	14 ± 2	23 ± 7		26 ± 9		22 ± 6
*Metrosideros excelsa*	20	20	1.4–12	7–51	4–47	163–463	25–41	20–40	510	17–188	889	9–40
			5.1 ± 3.1	24 ± 12	25 ± 11	243 ± 92	33 ± 3	26 ± 8		49 ± 53		23 ± 9
*Pittosporum crassifolium*	21	21	0.1–3	1–26	2–16	19–146	8–10	8–30	396	8–40	441	7–20
			0.8 ± 0.8	12 ± 7	7 ± 4	82 ± 34	9 ± 1	19 ± 5		21 ± 8		16 ± 5
*Pittosporum eugenioides*	19	19	0.1–0.7	2–25	2–6	4–33	8–14	15–20	370	15–40	469	11–22
			0.2 ± 0.1	8 ± 6	3 ± 1	16 ± 6	11 ± 2	19 ± 2		25 ± 9		18 ± 3
*Pittosporum tenuifolium*	18	18	0.02–1	0–27	1–6	40–97	4–9	10–20	258	10–40	340	10–28
			0.3 ± 0.2	9 ± 8	4 ± 1	66 ± 17	7 ± 1	14 ± 4		19 ± 10		14 ± 5
*Sophora microphylla*	21	21	0.7–13	6–53	5–72	338–1116	31–54	10–30	391	10–60	545	10–60
			7 ± 3	25 ± 13	34 ± 18	756 ± 213	46 ± 7	19 ± 5		26 ± 13		19 ± 10

**Table 3:** Overview of species’ mean site trait minima (‘min’) and maxima (‘max’) values, given are absolute values by species with the abbreviation of the respective site within brackets as follows: A, Auckland; C, Canterbury; D, Dunedin; H, Hawke’s Bay; N, Nelson-Tasman/Marlborough; T, Taranaki; and W, Wellington.

Species	Nectar															Flowers									
	Volume					Sugar					Concentration					Mass					Size				
	(µL)					(mg)					(°Brix)					(mg)					(mm)				
	min		-	max		min		-	max		min		-	max		min		-	max		min		-	max	
*Cordyline* *australis*	2	(H)	–	4	(D)	0.1	(A/T)	–	0.3	(D)	2	(A)	–	10	(H)	14	(T)	–	25	(H/W)	5	(N)	–	9	(D)
*Fuchsia* *excorticata*	12	(C)	–	34	(H)	2	(D)	–	5	(W)	8	(D)	–	20	(W)	12	(C)	–	314	(D)	14	(T)	–	26	(N)
*Leptospermum* *scoparium*	1	(N)	–	7	(D)	0.1	(H)	–	0.5	(D)	3	(H)	–	8	(D)	24	(H)	–	48	(W)	11	(H)	–	17	(N)
*Metrosideros* *excelsa*	10	(A)	–	35	(D)	3	(A)	–	11	(C)	12	(N)	–	43	(C)	176	(H)	–	423	(D)	30	(W)	–	38	(D)
*Pittosporum* *crassifolium*	4	(C)	–	14	(D)	0.2	(C)	–	2.5	(D)	6	(N)	–	20	(D)	19	(H)	–	118	(N)	8	(C)	–	9	(D)
*Pittosporum* *eugenioides*	3	(A)	–	6	(W)	0.1	(D)	–	0.4	(C/H)	3	(W)	–	16	(H)	8	(A)	–	27	(D)	9	(A)	–	13	(C)
*Pittosporum* *tenuifolium*	2	(H)	–	6	(A)	0.1	(A)	–	0.6	(T)	2	(A)	–	21	(T)	45	(H)	–	97	(T)	7	(D)	–	8	(C)
*Sophora* *microphylla*	20	(C)	–	54	(H)	3	(A)	–	9	(H)	9	(A)	–	42	(C)	573	(D)	–	866	(A)	37	(D)	–	52	(N)

Our study aimed to maximize reproducibility and accuracy in measuring nectar volumes while accounting for field conditions. Measuring environmentally available nectar (‘standing crop’) poses a significant challenge, as flowers may be depleted entirely due to prior foraging. While this approach is valuable for understanding plant-pollinator interactions, it does not provide reliable data for investigating physiological nectar production processes, which was our primary focus. The removal-refill method, where the standing crop is removed initially, and bagged flowers are left to refill nectar under controlled conditions for a day, was also unsuitable due to practical limitations. Repeated probing with micropipette tips can damage delicate flower tissues, particularly when sampling on moving branches in field conditions. Moreover, species with small flowers requiring rinsing cannot be sampled reliably while remaining on the plant.

Therefore, we opted for a non-invasive approach, accounting for partially foraged nectar as a baseline (the standing crop) while introducing a controlled 24-hour accumulation period. To exclude nectar feeders during this period, we covered flowers with transparent synthetic organza bags and used transparent rain shields when necessary. The following day, we cut the flower-bearing branch ends from the plants and sealed them in damp paper tissue. We then processed the flowers immediately, working sequentially through each species to minimize any effects from the water supply change. During processing, we removed the nectar, measured each flower’s maximum dimension, and weighed them.

First, we randomly removed 20–40 suitable flowers and extracted their nectar using micropipettes. Nectar was pooled into a pre-weighed 1.5 mL vial (Eppendorf, Germany) assigned to the respective tree. We collected pure nectar from species with larger flowers, such as pōhutukawa, kōwhai, and kōtukutuku. For species with smaller flowers, we rinsed the flowers with 5–20 µL of distilled water to collect their smaller nectar amounts (Morrant et al., 2009). We worked on a hydrophobic plastic sheet to collect nectar solution runoff when rinsing these flowers.

Nectar mass was measured as the difference in mass between the empty and filled vials using a balance (MS120, Mettler Toledo, Switzerland, accuracy 1 mg). To convert nectar mass into volume, we required the nectar’s specific density which we gained via converting its measured concentration. To determine the net nectar mass of rinsed flower samples, we adjusted for the volume of water added by subtracting its mass (1 µL ~ 1 mg) from the total mass of the pre-weighed vial. We then determined the resulting dilution factor by calculating the ratio of net nectar volume to the volume of water added.

Nectar concentrations were measured in °Brix using a digital refractometer (Atago PAL-1 3810, Japan; accuracy: 0.1°) with 20–100 µL of pure nectar or nectar solution obtained from rinsed flowers. Using the previously calculated dilution factors, we adjusted the measured Brix values of the nectar solutions to account for the water added during rinsing. Sugar mass was derived from the measured Brix value, assuming that 1° Brix corresponds to 100 g of sugar per liter of solution; accordingly, 100 g of nectar with a Brix of 10° contains approximately 10% dissolved solids, equivalent to 10 g of sugar. Any remaining nectar was frozen for future analysis.

**Figure 3:**
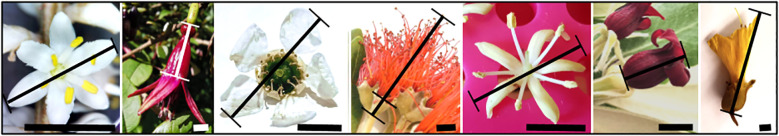
Methodology for measuring flower size per species, with the following species from left to right: tī kōuka *Cordyline australis*, kōtukutuku *Fuchsia excorticata*, mānuka *Leptospermum scoparium*, pōhutukawa *Metrosideros excelsa*, tarata *Pittosporum eugenioides*, karo *Pittosporum crassifolium*, also serving as a representative example for *P. tenuifolium*; *Sophora microphylla*. The scale bars indicate 5 mm.

Flower size was measured with digital calipers. The dimension was defined for each species (see **Figure 3**): corolla diameter was measured for mānuka and tī kōuka, and corolla length for kōwhai, kōtukutuku, and *Pittosporum* species. The flower size of pōhutukawa is the total longitudinal lengths of the flower, summing the lengths of the capsule and the mean lengths of 20 measured filaments per inflorescence.

Lastly, the mean flower mass was determined by weighing 20–40 flowers per tree using the same balance as for nectar mass and dividing their sum by the number of flowers weighed.

### 2.4 Data analysis

First, to evaluate general trends between plant traits across species, each species’ raw values (**Supplementary Tables S1a–h**) were standardized to a 0 to 1 scale and then pooled into a global dataset (n = 164 total samples). Plant traits included *i)* floral traits, such as flower fresh mass and flower size; and *ii)* nectar traits, such as volume, sugar amount, and concentration. The global dataset was then examined using linear regression analyses examining relationships between response variables (**Table 4**). Species-specific linear regression analyses (n = 18–27 samples/species) followed this investigating the consistency of intraspecific relationships between plant traits across species.

Second, to examine regional variation of each plant trait across species (n = 21–26 samples/region) using the global dataset, trait values initially underwent normality testing using Shapiro-Wilk tests and histograms. Based on the normality results, traits were then analyzed using either parametric tests (One-Way ANOVA with Tukey’s Honestly Significant Difference, HSD) or non-parametric tests (Kruskal-Wallis with Dunn’s test).

Third, we repeated this analysis species by species (~[3n/species]/site) to identify, whether intraspecific trends diverted from the global trends. Based on the small sample size of individuals per species per site, intraspecific results on regional variation have been treated with caution.

Fourth, due to the lack of uniformity of regional plant trait variation detected across species, the general global dataset was refined by excluding species showing no regional variation within the respective trait, after which our analysis was repeated. Additionally, we performed Principal Component Analyses (PCA) on scaled data for each species, including all five plant traits, to test for group differences by ‘climate zone’ (n = 5; Northern-North Island, Eastern-North Island, South Western-North Island, Northern-South Island, and Eastern-South Island), ‘coast’ (n = 2; east/west, with sites Auckland, Nelson-Tasman/Marlborough, Taranaki, and Wellington defined as ‘west’), and ‘New Zealand main island’ (n = 2; north/south). Due to the small sample size per site, it was not possible to calculate ellipses representing variation by ‘site’ itself.

Lastly, to assess the effects of climate and geographical explanatory variables (mean annual sunshine hours – MSH; mean annual additive rainfall amounts – MAR; mean annual air temperature – MAT; mean annual relative humidity – MRH; latitude; and longitude) on regional plant trait variation, correlations among predictor variables were initially identified using Pearson’s correlation analyses. Strong correlations between latitude, longitude, and climate variables led to the exclusion of geographical variables from the generalized additive mixed modeling (GAMM) to prevent multicollinearity. This GAMM analysis explored the relationship between climate variables and plant traits by modeling each response variable as a function of the predictors. We refined the models iteratively to retain only statistically significant predictors. The analysis was conducted on the reduced global dataset and separately for each species, with species treated as a categorical variable with eight levels.

Mean values are given as mean ± standard deviation throughout the text. All statistical analyses were performed using the R packages ‘MASS’, ‘mgcv’, ‘statmod’, ‘dplyr’, ‘ggplot2’, ‘png’, ‘grid’, ‘latex2exp’, ‘dunn.test’, and ‘tweedie’ within R versions 3.6.3 - 4.3.1 (R Core Team, 2020-2023).

## 3 Results

Across species (n = 8: tī kōuka *Cordyline australis*, kōtukutuku *Fuchsia excorticata*, mānuka *Leptospermum scoparium*, pōhutukawa *Metrosideros excelsa*, karo *Pittosporum crassifolium*, tarata *P. eugenioides*, kōhūhū *P. tenuifolium*, and kōwhai *Sophora microphylla*), we collected and weighed 4,276 individual flowers from 164 trees (see **Tables 1**–**3**, **Supplementary Tables S1a–h**).

We measured the size of 2,999 of these flowers and extracted nectar from 3,222, yielding a total of 2,240 µL of nectar. Trees (n = 3±1) representing every species were sampled at each site.

*Nectar Traits*: Flowers produced 3–34 µL of nectar/flower, containing 0.2–7mg of sugar/flower based on concentrations between 6–25°Brix (n = 21–26, based on the nectar of 258–510 flowers).

*Floral Traits:* Flowers had an average fresh mass between 19–756 mg (n = 340–889) and a size between 7–46mm (n = 245–465). All minima values were recorded for tī kōuka, all maxima for kōwhai.

Tī kōuka (n = 18 trees, see S1a) flowers (n = 440) had an average fresh mass between 14 mg (in Taranaki) – 25 mg (in Hawke’s Bay and Wellington), measured between 5 mm (in Nelson-Tasman/Marlborough) – 9 mm (in Dunedin) in diameter, and produced 2 µL (in Hawke’s Bay) – 4 µL (in Dunedin) of nectar, containing 0.1 mg (in Auckland and Taranaki) – 0.3 mg (in Dunedin) of sugar based on a Brix of 2° (in Auckland) – 10° (in Hawke’s Bay).

Kōtukutuku (n = 27, see S1b) flowers (n = 633) weighed between 12 mg (in Canterbury) – 314 mg (in Dunedin), measured between 14 mm (in Taranaki) – 26 mm (in Nelson-Tasman/Marlborough) in length, and produced 12 µL (in Canterbury) – 34 µL (in Hawke’s Bay) of nectar/flower, containing 2 mg (in Dunedin) – 5 mg (in Wellington) of sugar/flower based on a Brix of 8° (in Dunedin) – 20° (in Wellington) on average across sites.

Mānuka (n = 20, see S1c) flowers (n = 519) weighed between 24 mg (in Hawke’s Bay) – 48 mg (in W), measured between 11 mm (in Hawke’s Bay) – 17 mm (in Nelson-Tasman/Marlborough) in diameter, and produced 1 µL (in Nelson-Tasman/Marlborough) – 7 µL (in Dunedin) of nectar/flower, containing 0.1 mg (in Hawke’s Bay) – 0.5 mg (in Dunedin) of sugar/flower based on a Brix of 3° (in Hawke’s Bay) – 8° (in Dunedin) on average across sites.

Pōhutukawa (n = 20, see S1d) flowers (n = 889) weighed between 176 mg (in Hawke’s Bay) – 423 mg (in Dunedin), measured between 30 mm (in Wellington) – 38 mm (in Dunedin) in length, and produced 10 µL (in Auckland) – 35 µL (in Dunedin) of nectar/flower, containing 3 mg (in Auckland) – 11 mg (in Canterbury) of sugar/flower based on a Brix of 12° (in Nelson-Tasman/Marlborough) – 43° (in Canterbury) on average across sites.

Karo (n = 21, see S1e) flowers (n = 441) weighed between 19 mg (in Hawke’s Bay) – 118 mg (in Nelson-Tasman/Marlborough), measured between 8.3 mm (in Canterbury) – 9.2 mm (in Dunedin) in diameter, and produced 4 µL (in Canterbury) – 14 µL (in Dunedin) of nectar/flower, containing 0.2 mg (in Canterbury) – 2.5 mg (in Dunedin) of sugar/flower based on a Brix of 6° (in Nelson-Tasman/Marlborough) – 20° (in Dunedin) on average across sites.

Tarata (n = 19, see S1f) flowers (n = 469) weighed between 8 mg (in Auckland) – 27 mg (in Dunedin), measured between 9 mm (in Auckland) – 13 mm (in Canterbury) in diameter (n = 348), and produced 3 µL (in Auckland) – 6 µL (in Wellington) of nectar/flower, containing 0.1 mg (in Dunedin) – 0.4 mg (in Canterbury and Hawke’s Bay) of sugar/flower based on a Brix of 3° (in Wellington) - 16° (in Hawke’s Bay) on average across sites.

Kōhūhū (n = 18, see S1g) flowers (n = 340) weighed between 45 mg (in Hawke’s Bay) – 97 mg (in Taranaki), measured between 6.6 mm (in Dunedin) – 8.0 mm (in Canterbury) in diameter (n = 245), and produced 2 µL (in Hawke’s Bay) - 6 µL (in Auckland) of nectar/flower, containing 0.1 mg (in Auckland) – 0.6 mg (in Taranaki) of sugar/flower based on a Brix of 2° (in Auckland) - 21° (in Taranaki) on average across sites.

Kōwhai (n = 21, see S1h) flowers (n = 545) weighed between 573 mg (in Dunedin) – 866 mg (in Auckland), measured between 37 mm (in Dunedin) – 52 mm (in Nelson-Tasman/Marlborough) in diameter, and produced 20 µL (in Canterbury) – 54 µL (in Hawke’s Bay) of nectar/flower, containing 3 mg (in Auckland) – 9 mg (in Hawke’s Bay) of sugar/flower based on a Brix of 9° (in Auckland) - 42° (in Canterbury) on average across sites.

Across sites (n = 7: Auckland, Canterbury, Dunedin, Hawke’s Bay, Nelson-Tasman/Marlborough, Taranaki, and Wellington; see **Table 1**, **Supplementary Table S2a**), we removed nectar from 395–620 flowers and 21–26 trees. In Auckland, we processed 395 flowers from 22 trees; in Canterbury, 428 from 24; in Dunedin, 620 from 24; in Hawke’s Bay, 432 from 23; in Nelson-Tasman/Marlborough, 471 from 26; in Taranaki, 466 from 21; and in Wellington, 410 flowers from 24 trees.

*Nectar Traits:* Flowers from all eight species produced 11 µL (in Canterbury) – 16 µL (in Dunedin) of nectar/flower, containing 1.5 mg (in Auckland) – 2.7 mg (in Canterbury) of sugar/flower based on concentrations between 11° (in Auckland) – 17° Brix (in Canterbury) on average.

*Floral Traits:* Flowers had an average fresh mass between 154 mg (in Canterbury) – 199 mg (in Taranaki) and a size between 16 mm (in Canterbury) – 20 mm (in Nelson-Tasman/Marlborough). Overall (**Supplementary Table S2a**), we measured three out of five mean minima (flower mass, size, and nectar volume) and two maxima (sugar/flower and Brix) for samples from Canterbury.

Across climate zones (n = 5: Northern North Island zone, incl. Auckland; South-Western North Island zone, incl. Taranaki and Wellington; Eastern North Island zone, incl. Hawke’s Bay; Northern South Island zone, incl. Nelson-Tasman/Marlborough; Eastern South Island zone, incl. Canterbury and Dunedin; see **Table 1**, **Supplementary Table S2b**), we sampled 395 flowers from 22 trees within the Northern North Island zone; 876 flowers from 45 trees within the South-Western North Island zone; 432 flowers from 23 trees within the Eastern South Island zone; 471 flowers from 26 trees within the Northern South Island zone; and 1048 flowers from 48 trees within the Eastern South Island zone.

*Nectar Traits:* We detected 37.5% (3 out of 8 spp.) of species’ site mean minima and 50% of maxima in nectar volume in the Eastern South Island zone. We found 50% (4 out of 8 spp.) of species’ site mean minima of nectar sugar amounts in the Northern North Island zone and 50% of maxima in Eastern South Island zone. Similarly, we found 37.5% of nectar concentration minima in the Northern North Island zone and 50% of maxima in the Eastern South Island zone.

*Floral Traits*: 50% of flower mass minima were found in the Eastern South Island zone, with equally 37.5% of maxima in the South-Western North Island zone and Eastern South Island zone. 37.5% of flower size minima and 63% of flower size maxima were detected in the Eastern South Island zone. Overall (**Supplementary Table S2b**), we measured all of the five maxima for samples from the Eastern South Island climate zone.

Across islands (n = 2; North Island, South Island; see **Table 1**, **Supplementary Table S2c**), we sampled 1703 flowers from 90 trees from the North Island, and 1519 flowers from 74 trees from the South Island.

*Nectar Traits:* Nectar volume minima and maxima were equally distributed across islands. 75% (6 out of 8 spp.) of nectar sugar amount minima were detected for North Island sites, with equally 50% of maxima found for each island. 63% of nectar concentration minima were found at North Island sites, with equally 50% of maxima found for each island.

*Floral Traits:* North Island sites were responsible for 75% of flower mass minima (lowest species’ site mean based on averaged flower mass per tree) across all eight species (all except kōwhai and kōtukutuku), with flowers from Hawke’s Bay alone accounting for 50% or four species (Mānuka, pōhutukawa, karo, and kōhūhū). Flower mass maxima were equally distributed across islands; however, Dunedin (South Island) alone accounted for 37.5% of flower size maxima or three out of eight species (Kōtukutuku, pōhutukawa, and karo). Flower size minima showed a similar pattern, with each sampling site having one flower size minimum, except for Dunedin, which had two (Kōhūhū and kōwhai). Flower size maxima, however, were not detected at any North Island sites, with all maxima found at South Island sites. Overall (**Supplementary Table S2c**), we measured four out of five trait minima (all except nectar volume) at North Island sites, and one trait maximum (flower size) for flowers from the South Island. The remaining minima and maxima were equally distributed across islands.

### 3.1 Linear relationships between plant traits

#### 3.1.1 Relationships between plant traits across species

Linear regression analyses across all species using data standardized by species (see **Supplementary Tables S1a–h**) revealed a strong positive relationship between nectar sugar content and concentration (linear model, lm, *P* < 0.001, R² = 0.46, see **Table 4**). Additionally, we found a weak positive relationship between nectar sugar content and volume (lm, *P* < 0.001, R² = 0.18). However, nectar concentration and volume were not significantly correlated. Significant linear models were expected for these variables, as sugar quantities are derived directly from concentration and volume. Nonetheless, within species, variation in total nectar sugar was more strongly associated with changes in concentration than with volume, with concentration decreasing as volume increased. Additionally, linear regression identified very weak positive relationships between flower mass and nectar volume (lm, *P* < 0.001, R² = 0.06), as well as between flower mass and size (lm, *P* < 0.001, R² = 0.08). These weak relationships might reflect a non-linear, allometric link between floral and nectar traits—an avenue we did not explore as it was beyond the scope of our study.

**Table 4:** Statistical parameters of linear regression analysis for detecting relations among plant traits.

Plant trait	Nectar		Flowers	
	Concentration	Volume	Mass	Size
Nectar Sugar	R² = 0.46 (positive), *P* < 0.001	R² = 0.18 (positive), *P* < 0.001	–	–
Nectar Concentration	–	–	–	–
Nectar Volume	–	–	R² = 0.06 (positive), *P* < 0.001	–
Flower Mass	–	–	–	R² = 0.08 (positive), *P* < 0.001

#### 3.1.2 Relationships between plant traits within species

Repeating the linear regression analysis of standardized plant traits individually by species revealed more diverse results among species. We interpreted these findings with caution, given the low R² values observed in several cases. However, more concentrated nectar consistently contained larger sugar amounts (lm, *P* < 0.001, R² = 0.18–0.76, with two kōwhai outliers removed). Furthermore, for four species (Kōtukutuku, pōhutukawa, karo, and kōwhai), greater nectar volumes were associated with higher sugar content (lm, *P* < 0.01, R² = 0.35–0.70) and either higher (Kōtukutuku) or lower (Kōwhai and kōhūhū) nectar concentrations (lm, *P* < 0.05, R² = 0.10–0.40). Kōwhai and pōhutukawa were the only species in which flower size showed a significant linear relationship with flower mass, albeit weakly for kōwhai (Kōwhai: lm, *P* = 0.01, R² = 0.24; pōhutukawa: *P* < 0.001, R² = 0.76), once more, potentially reflecting a non-linear, allometric link between floral traits. We identified significant but weaker relationships between smaller mānuka flowers and greater nectar volumes (lm, *P* = 0.01, R² = 0.27); heavier kōtukutuku flowers, as well as heavier and larger kōwhai flowers and greater nectar volumes (lm, *P* < 0.05, R² = 0.20–0.32); and lastly, larger kōwhai flowers and lower nectar concentrations (lm, *P* < 0.01, R² = 0.27).

#### 3.1.3 Variation of plant traits within species across sites

Based on the small sample size per species per site, which also prevented us from calculating species-specific ellipses when testing for the grouping factor ‘sample site’ in Principal Component Analysis (PCA), the following results should be interpreted with caution. However, PCA was feasible on a species-by-species basis when testing for the groups ‘climate zone’ (n = 5; Northern North Island, Eastern North Island, South-Western North Island, Northern South Island, and Eastern South Island), ‘coast’ (n = 2; east/west), and ‘New Zealand main island’ (n = 2; north/south).

**Figure 4:**
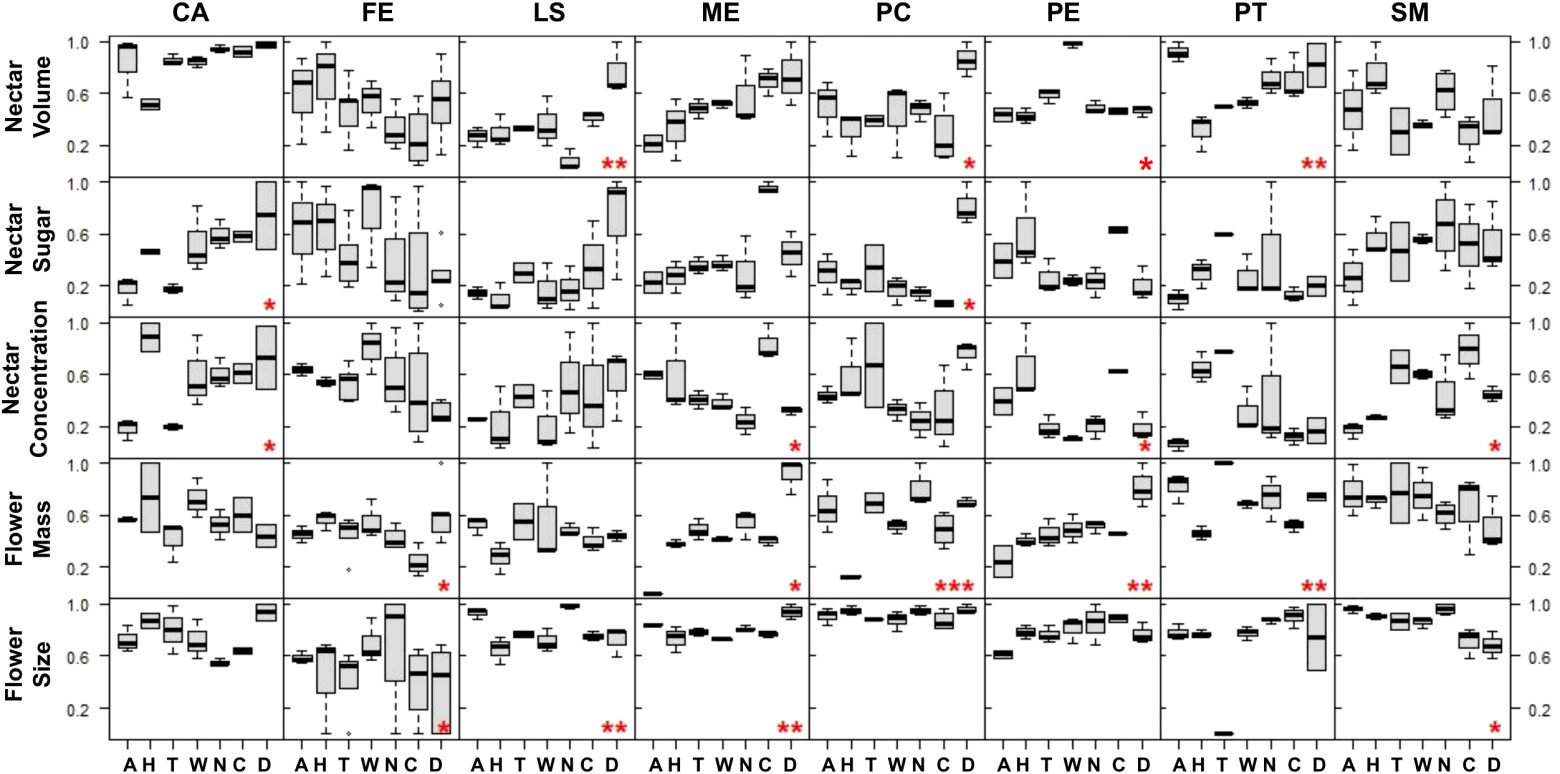
Standardized trait variation (rows) of the eight analyzed species (columns, with ‘CA’, *Cordyline australis*; ‘FE’, *Fuchsia excorticata*, ‘LS’, *Leptospermum scoparium*; ‘ME’, *Metrosideros excelsa*; ‘PC’, *Pittosporum crassifolium*; ‘PE’, *Pittosporum eugenioides*; ‘PT’, *Pittosporum tenuifolium*, ‘SM’, *Sophora microphylla*) across sites: Data presented in boxplots with black bars indicating region median values. Sites are ordered by latitude from left to right (north to south), with ‘A’, Auckland; ‘H’, Hawke’s Bay; ‘T’, Taranaki; ‘W’, Wellington; ‘N’, Nelson-Tasman/Marlborough; ‘C’, Canterbury; ‘D’, Dunedin. Red asterisks denote significant differences across sites: ‘*’ = *P* < 0.05, ‘**’ = *P* < 0.01, and ‘***’ = *P* < 0.001.

Individual PCA results revealed overlapping ellipses among all tested groups, indicating that plant trait compositions were not distinctly separated across these categories. Given the inherent sensitivity of PCA to sample size, particularly when estimating group variability, the observed overlapping ellipses may be influenced by the limited number of samples per site rather than an actual lack of distinction in trait composition. However, since all species exhibited significant plant trait variation by sample site in two to three of the five traits (**Table 5**, **Figure 4**), as demonstrated by parametric and non-parametric analyses, the observed ellipse overlap may be attributable to the limited sample size per site.

**Table 5:** Kruskal-Wallis (KW) and One-Way ANOVA (AOV) analysis results for the plant trait variation within species across sites.

Species	Nectar				Flowers			
	Sugar/ Flower	Concentration	Volume/ Flower	Samples	Mass		Size	
	*P*-value	*P*-value	*P*-value	n	*P*-value	n	*P*-value	n
*Cordyline australis*	0.02 (AOV)	<0.01 (AOV)	–	18	–	20–50	–	16–33
*Fuchsia excorticata*	–	–	–	27	0.01 (AOV)	6–50	0.01 (KW)	11–38
*Leptospermum scoparium*	–	–	< 0.01 (AOV)	20	–	19–40	< 0.01 (AOV)	18–40
*Metrosideros excelsa*	–	0.03 (KW)	–	20	0.03 (KW)	17–188	< 0.01 (KW)	9–40
*Pittosporum crassifolium*	0.03 (KW)	–	0.04 (AOV)	21	< 0.001 (AOV)	8–40	–	7–20
*Pittosporum eugenioides*	–	0.03 (KW)	0.04 (KW)	19	< 0.01 (AOV)	15–40	–	11–22
*Pittosporum tenuifolium*	–	–	< 0.01 (AOV)	18	< 0.01 (AOV)	10–40	–	10–28
*Sophora microphylla*	–	<0.01 (AOV)	–	21	–	10–60	< 0.01 (KW)	10–60

In particular, across sample sites, total sugar amounts significantly varied in only two species (Tī kōuka: ANOVA ‘aov’, *P* = 0.02; Karo: Kruskal-Wallis ‘KW’, *P* = 0.03), however only weakly. Nectar volumes (Mānuka and *Pittosporum* spp.; aov, KW, *P* = 0.04–0.009), concentrations (Tī kōuka, pōhutukawa, tarata, and kōwhai; aov, KW, *P* = 0.03–0.009), and flower sizes (Kōtukutuku, mānuka, pōhutukawa, and kōwhai; aov, KW, *P* = 0.01–0.009) varied each in four different species. Flower mass varied in five out of eight species (Kōtukutuku, pōhutukawa, *Pittosporum* spp., aov, KW, *P* = 0.03–0.0009). Therefore, total sugar amounts varied significantly between sample sites in the fewest number of species, while flower mass varied in the most.

In all species, except for tī kōuka, kōtukutuku, and mānuka, nectar volume and concentration varied inversely across sample sites (**Figure 4**), leading to similar total sugar amounts. This explains the low number of species with varying total sugar amounts per region and the low goodness of fit of our linear regression models for these plant traits. Furthermore, flower mass and size tended to vary with each other across sample sites (**Figure 4**); however, this relationship was only statistically significant for pōhutukawa. We could not identify a general visual trend between flower mass or size and nectar traits across sample sites based on the data displayed in **Figure 4**.

Most maxima for nectar sugar (75%) and flower size (100%) within species were observed in South Island sample sites (**Supplementary Table S2c**), despite this part of the country being represented by one fewer sample site (n = 3 versus n = 4 for North Island). Lastly, 65% of all ranked trait maxima were observed in the south, with 5% in Auckland (37°S) and 37.5% in Dunedin (45°S).

### 3.2 Environmental drivers of plant trait variation

As we detected strong correlations (Pearson’s correlation coefficients = 0.78–0.97; *P* < 0.001–0.04; **Table 6**) between geographical (latitude and longitude) and climate (mean annual rainfall ‘MAR’, mean annual air temperature ‘MAT’, mean annual relative humidity ‘MRH’, and mean annual sunshine hours ‘MSH’) variables in our analysis, geographical variables were excluded from the GAMM analysis.

**Table 6:** Pearson’s correlation matrix showing correlation coefficients and *P*-values for the correlations between environmental variables (bottom), with ‘MAR’, mean annual rainfall; ‘MAT’, mean annual air temperature in °C; ‘MRH’, mean annual relative humidity; ‘MSH’, mean annual sunshine hours; ‘LAT’, latitude; ‘LON’, longitude.

Environmental variables	Pearson’s correlation coefficients					*P*-values				
	MAR	MAT	MRH	MSH	LAT	MAR	MAT	MRH	MSH	,LAT
MAT	0.343					0.451				
MRH	0.436	-0.409				0.329	0.362			
MSH	-0.004	0.359	-0.363			0.993	0.429	0.424		
LAT	-0.426	**-0.968**	**0.306**	-0.491		0.34	**0**	0.505	0.263	
LON	0.108	**0.799**	**-0.797**	0.563	**-0.779**	0.818	**0.031**	**0.032**	0.188	**0.039**

Significant correlations are marked in bold.

#### 3.2.1 Correlations between plant traits and environmental factors across species

**Figure 5:**
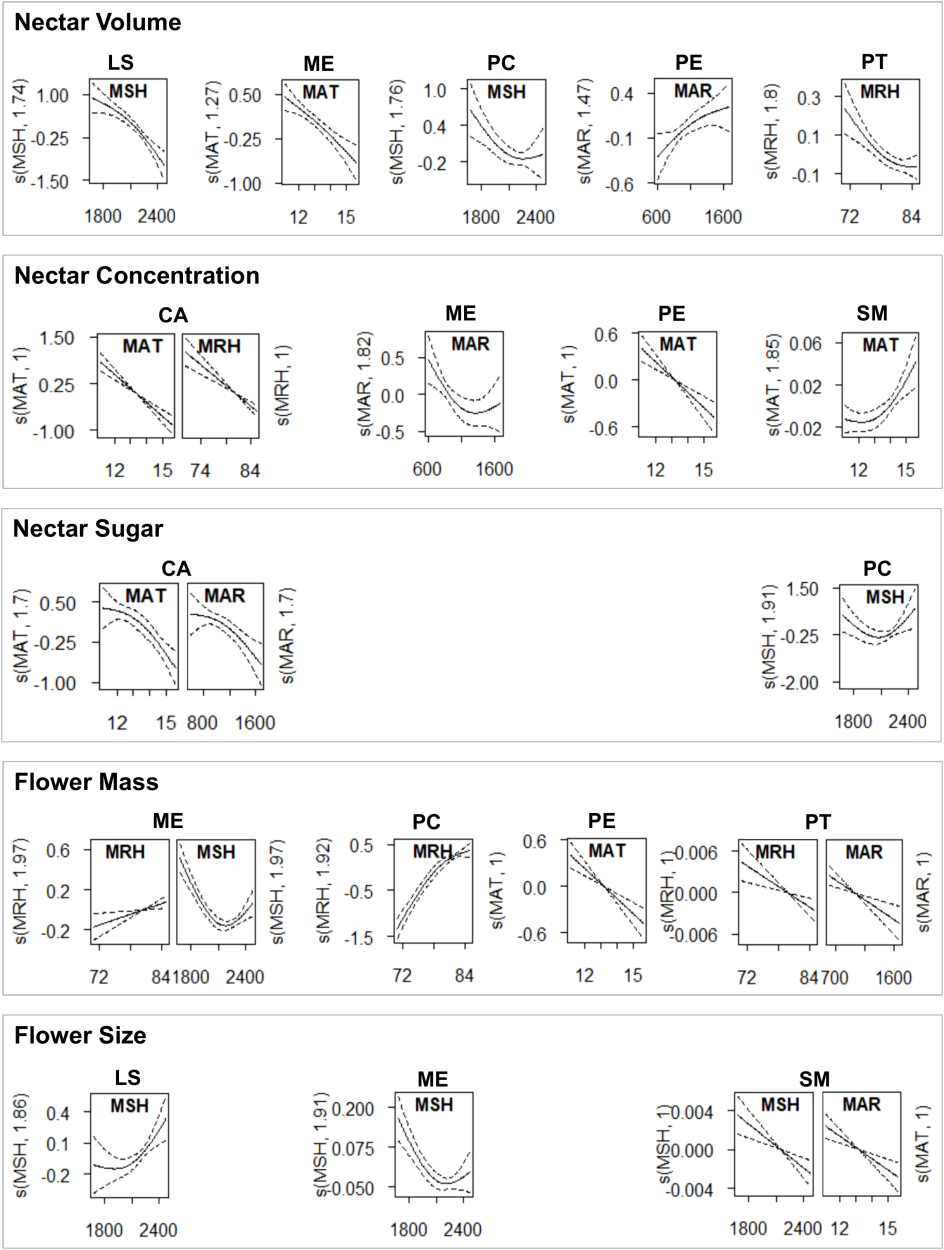
Significant intraspecific trait variations explained by climate factors, derived from the generalized additive mixed models (GAMM) with the best fit.

Based on the general global data (n = 164) of previously standardized data by species, only flower mass (KW, *P* = 0.04, R² = 13.2) and size (KW, *P* < 0.01, R² = 17.3) varied significantly between sites. In particular, flowers from Nelson-Tasman/Marlborough were larger than all others. Flowers from Nelson-Tasman/Marlborough, Wellington, and Dunedin were heavier than those from Hawke’s Bay and Canterbury (both flower traits: Dunn’s test *P* = 0.01–0.0006). MSH explained these regional variations very weakly (GAMM, flower size: *P* = 0.04, R² = 0.02; flower mass: *P* < 0.01, R² = 0.05). Given these weak results, we investigated whether a reduced dataset leads to stronger effect sizes, considering only a subset of species that previously showed significant regional variation (see **Figure 5**, **Table 1**).

**Figure 6:**
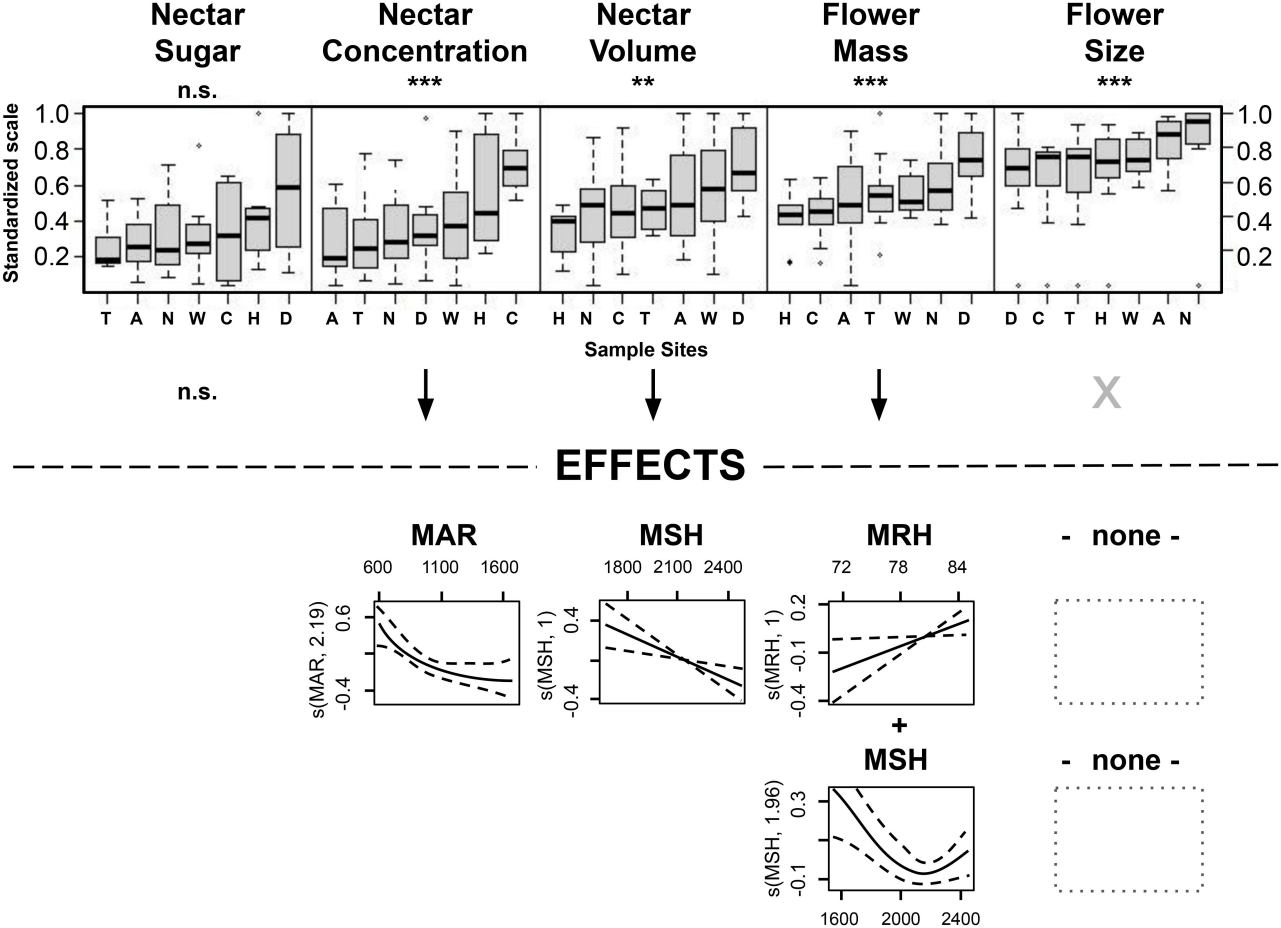
Variation in standardized data across sites, considering only species exhibiting significant differences for each plant trait, with data for boxplots (top of the figure) and GAMM (bottom of the figure, with GAMM shown only for significantly varying plant traits across sample sites) for sugar mass of the species tī kōuka and karo; for nectar concentration of tī kōuka, pōhutukawa, tarata and kōwhai; for nectar volume of mānuka and all *Pittosporum* species; for flower mass of kōtukutuku, pōhutukawa and all *Pittosporum* species; and flower size of kōtukutuku, mānuka, pōhutukawa, and kōwhai. Sample sites are labeled as in **Figure 4**, with each boxplot’s sample region order ranked by the y variable. The (partial) effects explain 14-23% of the variation. Asterisks below plant traits denote significance levels of the covariance: ‘*’ signifies *P* < 0.05, ‘**’ indicates *P* < 0.01, and ‘***’ denotes *P* < 0.001, with 'n.s.', not significant.

The reduced dataset for ‘nectar sugar’ comprised two species (Tī kōuka, karo, n = 38); ‘nectar concentration’ (Tı̄ kōuka, pōhutukawa, tarata, kōwhai; n = 78), ‘nectar volume’ (Mānuka, *Pittosporum* spp.; n = 78), and the ‘flower size’ (Kōtukutuku, mānuka, pōhutukawa, kōwhai; n = 88) comprised four species each, while ‘flower mass’ included five species (Kōtukutuku, pōhutukawa, *Pittosporum* spp., n = 105). All subsets, except for nectar sugar, demonstrated significant regional variations across species (**Table 7**, **Figure 6**). We detected a general trend of higher nectar concentrations in Canterbury and Hawke’s Bay, nectar volumes and flower masses in Dunedin, and larger flower sizes in Nelson-Tasman/Marlborough.

**Table 7:** Kruskal-Wallis analysis, Dunn’s test and GAMM analysis results for the effects of region and climate on standardized plant traits pooled for the species where significant regional differences were detected (subset of global data set).

Plant trait	n	Species	Kruskal-Wallis test	Dunn’s test	GAMM
Nectar Sugar	57	*Cordyline australis*, *Pittosporum crassifolium*	–	–	–
Nectar Concentration	78	*Cordyline australis*, *Metrosideros excelsa*, *Pittosporum eugenioides*, *Sophora microphylla*	R^2^ = 22.0, *P* = 0.001	*P* < 0.02	R² = 0.23, *P* < 0.01
Nectar Volume	78	*Leptospermum scoparium*, *Pittosporum* spp.	R^2^ = 17.8, *P* < 0.01	*P* < 0.01	R² = 0.14, *P* < 0.001
Flower Mass	105	*Fuchsia excorticata*, *Metrosideros excelsa*, *Pittosporum* spp.	R^2^ = 29.4, *P* < 0.001	*P* < 0.01	R² = 0.22, *P* < 0.05 (MRH), *P* < 0.01 (MSH)
Flower Size	88	*Fuchsia excorticata*, *Leptospermum scoparium*, *Metrosideros excelsa*, *Sophora microphylla*	R^2^ = 22.6, *P* = 0.001	*P* < 0.01	–

The higher nectar concentrations in Canterbury and Hawke’s Bay could partly be attributed to lower MAR (MAR < 790 mm) in those two eastern sites that lie in the rain shadow of axial mountain ranges. Moderate nectar concentrations were detected in Nelson-Tasman/Marlborough, Dunedin, and Wellington, sites with moderate mean annual rainfall amounts (959–1250 mm). The lowest concentrations were found in Auckland and Taranaki, regions that show moderate high to high annual rainfall amounts (1119–1683 mm).

In contrast, the higher nectar volumes and flower masses in Dunedin could be partially explained by the site’s lower MSH (Dunedin: 1681 h; other sites: 2062–2472 h) and, for flower mass additionally, the higher MRH (Dunedin: 85%; other sites: 71–82%, except T: 85%, with high MSH = 2197 mm), indicative of this southerly located sample site in this study. However, we could not identify any predictors that sufficiently explained the regional variance in flower size.

#### 3.2.2 Correlations between plant traits and environmental factors within species

Among all species, climate emerged as a key driver of trait variation, with MAR and MSH being the most influential factors (**Figure 5**, **Supplementary Table S3**). In general, nectar volume tended to decrease while nectar concentration increased in response to variables associated with evaporation or water availability (decreased MAR, increased MSH, or decreased MRH), although these effects varied among species. The climate responses of flower size and mass were even more species-dependent.

The type and extent of climate’s influence varied among species, with climate variables explaining variations in two to three traits across species, with deviances between 18% and 84%. Specifically, MAR affected all traits except flower size in at least one species. Similarly, depending on the species, MSH influenced all traits except nectar concentration; MAT impacted total sugar, nectar concentration, and flower size; MRH affected nectar volume, concentration, and flower mass.

The direction of regional climate effects was also variable: for nectar volume in four species, MAR had a positive effect, while MRH and MSH had negative impacts, with deviances from 18% to 64%. Nectar sugar in two species was negatively influenced by MAR and MAT, with deviances between 51% and 80%. Four species showed nectar concentration variations due to MAR, MRH (negative), or MAT (variable), with deviances from 29% to 58%. Flower mass in five species was influenced negatively by MAR or MSH or variable by MRH, with deviances between 54% and 85%. Lastly, flower size in four species was negatively affected by MAR or variable by MSH, with deviances ranging from 44% to 63%.

Among species, sunnier habitats typically resulted in larger kōtukutuku flowers but smaller ones for kōwhai, pōhutukawa, and tī kōuka. Warmer environments were linked to reduced sugar and concentration in tī kōuka nectar, decreased pōhutukawa nectar volume, and more concentrated nectar in kōwhai. However, air temperature did not directly influence flower mass or size. In rainier environments, tī kōuka nectar contained less sugar, pōhutukawa nectar was less concentrated, tarata flowers were larger but lighter, kōhūhū flowers were lighter, and kōwhai flowers were larger. More humid conditions generally increased tī kōuka nectar volume but decreased it in kōhūhū, and led to less concentrated nectar in both tī kōuka and tarata.

## 4 Discussion

Our analysis of the influence of climate on nectar and flower traits was based on long-term climate norms, allowing us to draw general conclusions about the traits of common New Zealand trees grown under diverse climate zone characteristics. These climatic factors impact trees not only during anthesis but throughout the entire year. While this approach may have masked potential effects of short-term meteorological or micrometeorological variations at the time of sampling or shortly beforehand—particularly relevant for nectar, a highly plastic trait—our broader approach allowed us to capture more general trends across species. However, these trends were shaped by diverse, species-specific patterns. This is understandable given the wide range of species included in our study, with only some being closely related. Consequently, our first hypothesis, proposing uniform relationships between climate drivers and plant traits across species, was only partially supported.

Across species, firstly, we observed a trend of higher nectar concentrations at New Zealand’s drier east coast sites, Canterbury and Hawke’s Bay (39.5–43.5°S, 172.6–176.8°E), which lie in the rain shadow of axial mountain ranges and consequently experience the lowest mean annual rainfall (MAR) across all sites (~600–800 mm compared to ~960–1700 mm). Secondly, nectar volumes and flower masses were generally greater at New Zealand’s most southerly east coast site, Dunedin (45.8°S, 170.5°E), which has the lowest mean annual sunshine (MSH) among all sites (~1700 hours compared to 2000+ hours) combined with one of the two highest mean annual relative humidity (MRH) values of 85%. Lastly, the sunniest of all analyzed sites and climate zones (~2500 hours annually; New Zealand’s ‘Northern South Island’ zone), including the regions Nelson-Tasman and Marlborough, tended to have the largest flowers. However, the exceptionally high annual sunshine hours did not emerge as the driving factor behind this trend, nor did any of the other tested climate variables, indicating the need for further investigation in future studies.

Within species, these general trends were not consistent, instead, this study revealed species-specific correlations between plant traits and climate factors in New Zealand tree species. Climate factors explained regional variation in different plant traits for each species, but the results varied in their degree of dependence and the direction of influence, as also described for other species by Plos et al. (2023). For instance, sugar amounts correlated positively with annual rainfall in tarata *Pittosporum eugenioides* but negatively in tī kōuka *Cordyline australis*. Regional variation in annual rainfall amounts only affected a single species’ nectar volume (positively), *Pittosporum eugenioides*, a correlation also observed in other species (e.g., Bertazzini and Forlani, 2016; Keasar et al., 2008).

Hypotheses 1a and 1b, which suggested (a) higher nectar volumes of (b) lower concentration in humid sites, were generally supported but not consistently observed within individual species. Across species (pooled data), the highest mean nectar volumes were observed in one of the two most humid sites, Dunedin. Within individual species, Dunedin had the highest nectar volumes from 50% (4 out of 8) of species. However, increased nectar volumes were more often associated with lower mean annual sunshine (MSH) rather than higher mean annual relative humidity (MRH). Conversely, we found generally higher nectar concentrations in New Zealand’s least humid sites, Canterbury and Hawke’s Bay, supporting hypothesis 1b.

An Australian study by Hawkins et al. (2018) also identified regional differences in nectar volumes, with solar radiation and precipitation as key drivers. These regional variations might arise from species or population-specific differences in compensatory mechanisms mitigating water stress effects (Iqbal et al., 2012; Balducci et al., 2016; Teixido et al., 2022), possibly due to past selection pressures induced by drought (Hawkins et al., 2018). However, the impact of drought—here particularly referring to low MAR and MRH (soil moisture was not investigated)—on nectar volume and concentration varies across studies, with possible negative (Descamps et al., 2021), positive (Suni et al., 2020), or neutral (Phillips et al., 2018) responses to water limitations. These limitations may be more prominent in arid sites relative to sites in New Zealand with little seasonal variation in MAR and with only modest moisture stress (Kidson, 2000).

Hypothesis 1c proposed that nectar sugar mass, flower fresh mass, and size would be highest in sites with abundant sunshine. Again, this hypothesis could only be partly confirmed. In the sunniest region, Nelson-Tasman/Marlborough, the presence of the largest flowers within 75% of species that showed regional variance (n = 4) supported this hypothesis. The positive correlation between larger flowers and sunnier sites may result from underlying genetic factors. Previous genetic studies have demonstrated that variations in corolla length, which we categorized as ‘flower size’ for tube- and bell-shaped flowers, within populations can be heritable. Genetic differences among individuals can account for the variation in corolla length (Galen, 1999). Therefore, it would be intriguing to investigate the extent to which the differences in flower size observed among the analyzed species across sites are also genetically influenced, an aspect not covered in this study.

On the other hand, sugar quantities hardly varied across sites, and variation in flower fresh mass was more closely linked to relative humidity than sunshine, with Dunedin consistently hosting the heaviest flowers. This unexpected pattern might stem from accelerated desiccation effects in sunnier sites, leading to uncontrolled water loss from flowers (Bourbia et al., 2020) and, hence, reduced fresh mass.

Global climate change is likely to alter the nectar production in our seven analyzed species. Rising temperatures will likely increase *Sophora microphylla* nectar concentration, resulting in higher viscosity, while reducing pōhutukawa *Metrosideros excelsa* nectar volume and lowering *Cordyline australis* and *Pittosporum eugenioides* nectar concentrations. In high-rainfall regions, such as New Zealand’s South-West North Island climate zone, increased precipitation may decrease *Metrosideros excelsa* nectar concentration and *Cordyline australis* sugar content, while enhancing *Pittosporum eugenioides* nectar volume. Higher relative humidity in these areas may further reduce *Cordyline australis* nectar sugar concentration and suppress kōhūhū *Pittosporum tenuifolium* nectar production. Conversely, rainshadow regions, including the east coast climate zones of both islands, will experience higher temperatures without increased rainfall, leading to lower humidity and more frequent droughts. These conditions are expected to reduce *Pittosporum eugenioides* nectar secretion, while increasing *Metrosideros excelsa* and *Cordyline australis* nectar concentration and viscosity due to increased evaporation and the plant’s reduced water status. Shifts in nectar viscosity could disrupt pollinator interactions. Highly viscous nectar may become inaccessible to sucking pollinators, such as bees, butterflies, moths, and flies, whose proboscis may be ineffective in extracting it. Conversely, reduced viscosity may deter other pollinators if the energy cost of nectar collection outweighs the sugar reward, necessitating more frequent flights. These changes in nectar properties may have cascading effects on pollination networks, potentially reshaping ecosystem dynamics.

In general, nectar and floral traits were positively correlated within species; larger and heavier flowers tended to produce higher volumes of nectar with greater sugar content. Nectar concentration was an important predictor of total nectar sugars, more so than nectar volume, suggesting that some of the observed intraspecific variation in nectar properties was associated with evaporation effects and plant water status. This has been observed in other species, such as *Silphium perfoliatum* (Mueller et al., 2020), mānuka *Leptospermum scoparium* (Clearwater et al., 2018), and *Epilobium angustifolium* (Bertsch, 1983), and not observed in other species, such as *Ipomopsis longiflora* (Villarreal and Freeman, 1990). At the interspecific level, nectar concentration increased with nectar volume, while nectar volume and total sugar amounts were not correlated with flower size or mass across species, indicating that evolutionary differences were less significant at the species level. While there were consistent correlations between traits within species, not all relationships were observed in every species.

Consequently, our second hypothesis was not supported, as it posited that correlations between plant traits would follow a consistent pattern within all species. For example, significant correlations between most pairs of traits were specific to individual species: higher nectar volumes containing more sugar were only observed in half of the tested species (kōtukutuku *Fuchsia excorticata*, *Metrosideros excelsa*, karo *Pittosporum crassifolium*, and kōwhai *Sophora microphylla*). Variation in trait relationships between species across individuals and sites may be linked to differences in nectar secretion processes, flower structure, or evolutionary relationships with pollinators (Vesprini et al., 1999; Pacini et al., 2003; Thompson et al., 2017). The latter has been observed in plant species adapted to specialized bird pollinators in West-Central Africa (Janeček et al., 2021). These plants demonstrated a positive correlation between nectar volume and total sugars, potentially aligning with the preferences and nutritional requirements of their avian pollinators. Although *Pittosporum crassifolium* is classified as an entomophilous species, it also attracts bird visits. The flowers of this tree can adequately fulfill the energetic requirements of even the largest New Zealand honeyeater, the tūi (*Prosthemadura novae-zealandiae*) (Castro and Robertson, 1997).

Karo’s sister taxon, *Pittosporum tenuifolium*, was the only species with a negative correlation between nectar volume and concentration. The contrasting relationships in nectar traits between *Pittosporum crassifolium* and *Pittosporum tenuifolium* may reflect adaptations to different pollinator assemblages. Both species attract birds and insects during the day, but *Pittosporum tenuifolium* may also draw nocturnal insects, such as moths, as its sweet fragrance intensifies at night (Allan, 1961; Wardle, 2011).

The lack of a strong interspecific correlation between standardized flower size and mass may be explained by flower mass reflecting not only size but also the thickness and tissue density of structures. This could be an adaptation to benefit floral longevity by conserving water in thicker and heavier petals, which do not necessarily need to be longer. These traits, such as more layers of petal cells or a thicker mesophyll (Guo et al., 2023), may contribute to floral longevity. The lack of correlation between flower size and mass of Auckland flowers may be due to the vastly different flower anatomies of our sampled species. We can conclude that flower mass and size are less important drivers of nectar variables within species than among species.

Our findings enhance understanding of local nectar production and its regional variation, with applications in both conservation and commercial contexts. For instance, estimates of regional nectar volume and floral biomass can inform assessments of food availability for nectar- and flower-feeding animals, including native and honey bees, as well as endemic birds.

In future landscape-scale studies, species-specific values for average flower size and flower number per tree can be combined to estimate total nectar volume. For *Fuchsia excorticata*, *Metrosideros excelsa*, *Pittosporum eugenioides*, and *Sophora microphylla*, such estimates do not require adjustments for regional climatic differences between New Zealand’s North and South Islands, as relative nectar volumes are consistent across regions.

However, for *Cordyline australis*, *Leptospermum scoparium*, *Pittosporum crassifolium*, and *Pittosporum tenuifolium*, regional variation must be taken into account. These species produce higher nectar volumes in the South Island, particularly in the Otago–Dunedin region, with *Pittosporum tenuifolium* also showing significantly high nectar volumes in Auckland.

When estimating nectar quality, north–south differences in sugar content are generally negligible, except for *Cordyline australis* and *Pittosporum crassifolium*, which produce higher sugar concentrations in southern populations.

For future studies focused on predicting food availability for flower-feeding birds, it is noteworthy that O’Donnell & Dilks (1994) recorded seven indigenous species primarily feeding on kōtukutuku flowers, constituting approximately 50% of these birds’ flower-based diets. Kōtukutuku flowers are largest in terms of length in New Zealand’s sunniest regions, Nelson-Tasman and Marlborough, suggesting a potential for greater energy intake with less foraging effort for flower-feeding birds. Notably, kōtukutuku exhibits a higher flower fresh mass in regions such as Hawke’s Bay, Wellington, and Otago.

While we lack data on the flower’s dry weight, this difference could be attributed to a higher water content within the flower tissue. This increased water content may benefit birds during the drier seasons, especially in the Hawke’s Bay region, which has high vapor pressure deficit conditions and low annual rainfall amounts (Kidson, 2000).

Our study provides a novel comparative framework for assessing how floral nectar traits vary within and among eight common endemic species across climatically diverse regions of New Zealand. By identifying which species exhibit consistent versus variable nectar production across environments, we contribute to a deeper understanding of how plants mediate interactions with climate through potential resource allocation strategies. However, the tested environmental factors explain only part of the observed regional variation, suggesting that additional influences—such as underlying genetic differences—may also contribute to within-species variability. We therefore recommend that future research investigates whether the sampled populations have diverged into distinct local genotypes. If such genetic differentiation is confirmed, we propose testing multiple genotypes per species under controlled environmental conditions to clarify the relative contributions of genetic and environmental factors to nectar trait variation.

## 5 Conclusion

This study contributes a novel comparative dataset spanning eight endemic New Zealand species across climatically diverse regions. By systematically quantifying five key nectar traits and linking them to regional climate variables, we provide a new framework for understanding species-specific patterns of nectar variation across landscapes. Importantly, we distinguish species whose nectar output is relatively stable across regions from those with strong climate-dependent responses—an innovation that improves our ability to model floral resource availability under changing environmental conditions. This comparative approach, combining ecological and practical metrics (nectar volume, sugar amount, flower size), represents a step toward more accurate predictions of how plant-pollinator interactions may respond to climate variability and change.

While a uniform pattern across species was not apparent, certain trends were evident: in sunnier sites, nectar volumes were generally lower, and flower sizes larger (as in Plos et al., 2023), whereas nectar concentrations tended to be higher in drier areas. Among all traits and across all species, standardized total sugar amounts varied the least between sites and in response to climate, confirming findings by Noe et al. (2019) from mānuka *Leptospermum scoparium*. Hence, the energetic value of nectar, arguably the most important trait for pollinators, is more predictable across sites, based on flower number and species alone, than the remaining four tested variables. Our observations will benefit the honey industry and conservation efforts, as our results could be used to provide estimates of nectar sugar availability based on flower numbers alone or flower number and size, depending on the species. However, with predicted global climate shifts, we can expect not only changes in nectar quantity and quality but also alterations in flower abundance, pollinator behavior, and, consequently, the structure of plant-pollinator interactions and ecosystem functioning. We recommend additional analyses considering factors such as genotype and soil nutrient availability to better comprehend other contributing drivers for nectar variation across regions. Additionally, assessing regional variation in species-specific flower abundance could enhance global change models for predicting nectar availability. For this, we recommend incorporating less, ideally closely related species, with ten or more samples per species per region. Ideally, microclimate variables would be measured for a standardized number of consecutive days before sampling to capture the specimen’s local conditions.

## Data Availability

The raw data supporting the conclusions of this article will be made available by the authors, without undue reservation.
