# Supplementary material for: Nectar traits of New Zealand trees vary across climatic zones
Source: Front Plant Sci. 2025 Oct 3;16:1539875. doi: 10.3389/fpls.2025.1539875 (PMC12531208; doi:10.3389/fpls.2025.1539875)
Supplement: Supplementary file 1 [file DataSheet1.docx]

**Supplementary Material** for the Manuscript of:

**Nectar Traits of New Zealand Trees vary across Climatic Zones**

**Johanna M van Delden, Sebastian Leuzinger, Sarah J Richardson, and Michael J Clearwater**

# List of Abbreviations (in alphabetical order)

A – Auckland;

C – Canterbury;

CA – tī kōuka (*Cordyline australis* Forster);

D – Dunedin;

FE – kōtukutuku *(Fuchsia excorticata* L f);

H – Hawke’s Bay;

LS – mānuka (*Leptospermum scoparium* J R Forst. & G Forst.);

ME – pōhutukawa *(Metrosideros excelsa* A Cunn. ex G Don);

MAR – mean annual additive rainfall amounts;

MAT – mean annual air temperature;

MRH – mean annual relative humidity;

MSH – Mean annual sunshine hours;

N – Nelson-Tasman/Marlborough;

PC – karo *(Pittosporum crassifolium* Banks & Sol.);

PE – tarata *(Pittosporum eugenioides* A Cunn.);

PT – kōhūhū *(Pittosporum tenuifolium* Gaertn.);

SM – kōwhai (*Sophora microphylla* Aiton);

T – Taranaki;

W – Wellington;

# Supplementary Tables

**Table S1a-h**: Sampling details from all species across all sites, with **a)** *Cordyline australis*, **b)** *Fuchsia excorticata*, **c)** *Leptospermum scoparium*, **d)** *Metrosideros excelsa*, **e)** *Pittosporum crassifolium*, **f)** *Pittosporum eugenioides*, **g)** *Pittosporum tenuifolium*, and **h)** *Sophora microphylla*.

**a)** *Cordyline australis* (n =18)

| **Site** | **Date** | **Tree** | **Flowers** | | | | **Nectar** | | | | |
| --- | --- | --- | --- | --- | --- | --- | --- | --- | --- | --- | --- |
|  |  |  | **Mean**  **Flower Mass** | | **Mean**  **Flower Size** | | **Total**  **Nectar Volume** | | **Mean Nectar**  **Volume / Flower** | **Mean**  **Sugar / Flower** | **Nectar Concentration** |
|  |  |  |  |  |  |  |  |  |  |  |  |
|  |  |  |  |  |  |  |  |  |  |  |  |
|  | (Month/Year) | ID | (mg) | n | (mm) | n | (µL) | n | (µL) | (mg) | (Brix) |
|  |  |  |  |  |  |  |  |  |  |  |  |
| A | 10/2020 | AD | 20 | 20 | 6.36 | 19 | 79 | 20 | 3.96 | 0.112 | 2.9 |
| A | 10/2020 | AE | 19 | 20 | 5.83 | 21 | 46 | 20 | 2.29 | 0.026 | 1.1 |
| A | 10/2020 | AF | 19 | 20 | 7.66 | 20 | 78 | 20 | 3.89 | 0.099 | 2.6 |
| C | 11/2020 | CHA2 | 16 | 20 | 6.06 | 19 | 77 | 20 | 3.87 | 0.238 | 6.3 |
| C | 11/2020 | CHZ | 25 | 20 | 5.65 | 19 | 71 | 20 | 3.54 | 0.274 | 8.0 |
| D | 11/2020 | DG2 | 12 | 20 | 7.96 | 20 | 77 | 20 | 3.83 | 0.214 | 5.7 |
| D | 11/2020 | DH2 | 18 | 20 | 9.18 | 20 | 81 | 20 | 4.03 | 0.440 | 11.4 |
| H | 10/2020 | NAG2 | 16 | 20 | 7.42 | 20 | 45 | 20 | 2.23 | 0.197 | 9.1 |
| H | 10/2020 | NAH2 | 34 | 20 | 8.54 | 20 | 38 | 20 | 1.91 | 0.213 | 11.7 |
| N | 11/2020 | ND2 | 22 | 20 | 5.36 | 17 | 78 | 20 | 3.90 | 0.250 | 6.6 |
| N | 11/2020 | NE2 | 14 | 20 | 4.95 | 21 | 75 | 20 | 3.76 | 0.312 | 8.6 |
| N | 11/2020 | NF2 | 18 | 20 | 4.84 | 20 | 73 | 20 | 3.67 | 0.215 | 6.0 |
| T | 12/2019 | NPY | 17 | 20 | 5.62 | 18 | 67 | 20 | 3.35 | 0.076 | 2.3 |
| T | 12/2019 | NPZ | 8 | 20 | 9.02 | 20 | 67 | 20 | 3.35 | 0.067 | 2.0 |
| T | 12/2019 | NPA1 | 17 | 20 | 7.32 | 20 | 73 | 20 | 3.64 | 0.093 | 2.6 |
| W | 11/2019 | W14 | 24 | 40 | 5.34 | 22 | 52 | 15 | 3.44 | 0.145 | 4.3 |
| W | 11/2019 | W15 | 20 | 50 | 8.12 | 16 | 53 | 15 | 3.56 | 0.361 | 10.6 |
| W | 11/2019 | W16 | 30 | 50 | 6.31 | 15 | 48 | 15 | 3.22 | 0.189 | 6.0 |

**b)** *Fuchsia excorticata* (n = 27)

| **Site** | **Date** | **Tree** | **Flowers** | | | | **Nectar** | | | | |
| --- | --- | --- | --- | --- | --- | --- | --- | --- | --- | --- | --- |
|  |  |  | **Mean Flower Mass** | | **Mean Flower Size** | | **Total**  **Nectar Volume** | | **Mean Nectar Volume / Flower** | **Mean**  **Sugar / Flower** | **Nectar Concentration** |
|  |  |  |  |  |  |  |  |  |  |  |  |
|  |  |  |  |  |  |  |  |  |  |  |  |
|  | (Month/Year) | ID | (mg) | n | (mm) | n | (µL) | n | (µL) | (mg) | (Brix) |
|  |  |  |  |  |  |  |  |  |  |  |  |
| A | 10/2020 | AP | 236 | 20 | 17.50 | 20 | 652 | 20 | 32.62 | 4.86 | 14.9 |
| A | 10/2020 | AQ | 263 | 40 | 15.65 | 38 | 827 | 20 | 41.33 | 7.11 | 17.2 |
| A | 10/2020 | AR | 201 | 20 | 15.05 | 20 | 195 | 20 | 9.74 | 1.55 | 15.9 |
| C | 11/2019 | C1 | 109 | 50 | 10.12 | 20 | 129 | 21 | 6.14 | 0.37 | 6.0 |
| C | 11/2019 | C2 | 68 | 30 | 17.78 | 13 | 22 | 11 | 2.02 | 0.04 | 2.0 |
| C | 11/2019 | C3 | 198 | 13 | 15.61 | 16 | 166 | 6 | 27.64 | 6.91 | 25.0 |
| C | 11/2019 | C5 | 109 | 50 | NA | NA | 68 | 5 | 13.68 | 1.78 | 13.0 |
| D | 9/2020 | DA | 198 | 20 | 12.35 | 20 | 858 | 20 | 42.92 | 4.33 | 10.1 |
| D | 9/2020 | DB | 312 | 20 | 18.76 | 18 | 659 | 20 | 32.96 | 2.24 | 6.8 |
| D | 9/2020 | DC | 512 | 35 | 17.41 | 20 | 920 | 35 | 26.29 | 1.58 | 6.0 |
| D | 9/2020 | DA2B | 310 | 10 | NA | NA | 351 | 20 | 17.56 | 1.70 | 9.7 |
| D | 9/2020 | DC2B | 239 | 25 | NA | NA | 148 | 25 | 5.90 | 0.35 | 6.0 |
| H | 10/2020 | NAV | 309 | 15 | 18.98 | 16 | 474 | 10 | 47.42 | 6.83 | 14.4 |
| H | 10/2020 | NAW | 249 | 6 | NA | NA | 231 | 6 | 38.58 | 4.98 | 12.9 |
| H | 10/2020 | NAX | 316 | 19 | 17.44 | 19 | 273 | 19 | 14.37 | 1.91 | 13.3 |
| N | 10/2019 | NEL2 | 277 | 6 | 27.52 | 23 | 158 | 6 | 26.30 | 6.29 | 23.9 |
| N | 10/2019 | NEL4 | 215 | 20 | 27.37 | 33 | 254 | 20 | 12.69 | 1.54 | 12.1 |
| N | 10/2019 | NEL6 | 180 | 8 | 22.68 | 21 | 65 | 8 | 8.15 | 0.64 | 7.9 |
| N | 10/2019 | NEL23 | 186 | 44 | NA | NA | 587 | 44 | 13.35 | 1.70 | 12.7 |
| T | 11/2020 | NPJ | 261 | 27 | 16.73 | 13 | 699 | 27 | 25.88 | 2.67 | 10.3 |
| T | 11/2020 | NPO | 287 | 13 | 15.48 | 11 | 475 | 13 | 36.52 | 5.55 | 15.2 |
| T | 11/2020 | NPP | 275 | 11 | 9.64 | 21 | 282 | 11 | 25.67 | 3.65 | 14.2 |
| T | 11/2020 | NPQ | 92 | 30 | 14.49 | 13 | 238 | 30 | 7.94 | 1.40 | 17.6 |
| T | 11/2020 | NPR | 219 | 15 | NA | NA | 249 | 15 | 16.57 | 1.66 | 10.0 |
| W | 11/2019 | W17 | 371 | 36 | 17.19 | 20 | 548 | 20 | 27.41 | 6.80 | 24.8 |
| W | 9/2020 | WP | 247 | 10 | 24.45 | 11 | 296 | 9 | 32.88 | 6.94 | 21.1 |
| W | 9/2020 | WQ | 232 | 40 | 15.76 | 20 | 496 | 31 | 15.99 | 2.40 | 15.0 |

**c)** *Leptospermum scoparium*

| **Site** | **Date** | **Tree** | **Flowers** | | | | **Nectar** | | | | |
| --- | --- | --- | --- | --- | --- | --- | --- | --- | --- | --- | --- |
|  |  |  | **Mean Flower Mass** | | **Mean Flower Size** | | **Total**  **Nectar Volume** | | **Mean Nectar Volume / Flower** | **Mean**  **Sugar / Flower** | **Nectar Concentration** |
|  |  |  |  |  |  |  |  |  |  |  |  |
|  |  |  |  |  |  |  |  |  |  |  |  |
|  | (Month/Year) | ID | (mg) | n | (mm) | n | (µL) | n | (µL) | (mg) | (Brix) |
|  |  |  |  |  |  |  |  |  |  |  |  |
| A | 10/2020 | AG | 39 | 19 | 16.35 | 19 | 52 | 20 | 2.62 | 0.090 | 3.7 |
| A | 10/2020 | AH | 48 | 20 | 15.13 | 20 | 34 | 20 | 1.68 | 0.060 | 3.9 |
| A | 10/2020 | AI | 48 | 20 | 16.50 | 19 | 63 | 20 | 3.15 | 0.120 | 3.8 |
| C | 11/2020 | CHF2 | 29 | 20 | 12.76 | 18 | 64 | 20 | 3.18 | 0.440 | 14.6 |
| C | 11/2020 | CHG2 | 43 | 20 | 13.56 | 20 | 83 | 20 | 4.13 | 0.210 | 5.2 |
| C | 11/2020 | CHH2 | 32 | 20 | 12.40 | 21 | 81 | 20 | 4.06 | 0.020 | 0.5 |
| D | 01/2020 | D11 | 39 | 40 | 13.54 | 40 | 245 | 40 | 6.12 | 0.630 | 10.8 |
| D | 01/2020 | D12 | 35 | 40 | 13.60 | 40 | 236 | 40 | 5.90 | 0.580 | 10.3 |
| D | 01/2020 | D13 | 41 | 20 | 10.11 | 20 | 370 | 40 | 9.25 | 0.160 | 3.5 |
| H | 12/2020 | NAO | 34 | 20 | 12.85 | 19 | 39 | 20 | 1.96 | 0.140 | 7.5 |
| H | 12/2020 | NAY2 | 13 | 20 | 9.13 | 20 | 45 | 20 | 2.26 | 0.030 | 1.6 |
| H | 12/2020 | NAZ2 | 26 | 20 | 11.58 | 18 | 81 | 20 | 4.06 | 0.020 | 0.5 |
| N | 9/2020 | NX | 46 | 40 | 17.20 | 20 | 7 | 20 | 0.33 | 0.010 | 2.2 |
| N | 9/2020 | NY | 39 | 40 | 16.96 | 20 | 7 | 20 | 0.37 | 0.100 | 13.6 |
| N | 9/2020 | NZ | 40 | 40 | 16.56 | 20 | 33 | 20 | 1.67 | 0.220 | 6.8 |
| T | 11/2020 | NPA5 | 59 | 20 | 12.77 | 19 | 65 | 20 | 3.25 | 0.240 | 7.6 |
| T | 11/2020 | NPA6 | 36 | 20 | 13.56 | 20 | 58 | 20 | 2.91 | 0.140 | 5.1 |
| W | 12/2020 | WR | 29 | 20 | 10.94 | 20 | 58 | 20 | 2.91 | 0.020 | 0.9 |
| W | 12/2020 | WS | 29 | 20 | 11.77 | 20 | 107 | 20 | 5.33 | 0.060 | 1.3 |
| W | 12/2020 | WT | 86 | 40 | 14.03 | 20 | 36 | 20 | 1.82 | 0.240 | 6.9 |

**d)** *Metrosideros excelsa*

| **Site** | **Date** | **Tree** | **Flowers** | | | | **Nectar** | | | | |
| --- | --- | --- | --- | --- | --- | --- | --- | --- | --- | --- | --- |
|  |  |  | **Mean Flower Mass** | | **Mean Flower Size** | | **Total**  **Nectar Volume** | | **Mean Nectar Volume / Flower** | **Mean**  **Sugar / Flower** | **Nectar Concentration** |
|  |  |  |  |  |  |  |  |  |  |  |  |
|  |  |  |  |  |  |  |  |  |  |  |  |
|  | (Month/Year) | ID | (mg) | n | (mm) | n | (µL) | n | (µL) | (mg) | (Brix) |
|  |  |  |  |  |  |  |  |  |  |  |  |
| A | 12/2019 | A32 | NA | NA | 34.20 | 33 | 280 | 40 | 7.00 | 1.828 | 29.0 |
| A | 12/2019 | A31 | NA | NA | 34.32 | 38 | 260 | 20 | 13.00 | 3.694 | 32.0 |
| C | 11/2020 | CHC2 | 168 | 20 | 30.47 | 11 | 540 | 20 | 27.00 | 11.144 | 51.0 |
| C | 11/2020 | CHD2 | 198 | 20 | 31.53 | 9 | 740 | 20 | 37.00 | 12.040 | 38.0 |
| C | 11/2020 | CHE2 | 196 | 20 | 31.80 | 20 | 680 | 20 | 34.00 | 11.107 | 39.0 |
| D | 01/2020 | D8 | 350 | 100 | 35.84 | 40 | 1880 | 40 | 47.00 | 7.406 | 17.0 |
| D | 01/2020 | D9 | 463 | 188 | 38.25 | 40 | 960 | 40 | 24.00 | 3.265 | 15.0 |
| D | 01/2020 | D10 | 456 | 172 | 40.82 | 40 | 1320 | 40 | 33.00 | 5.498 | 18.0 |
| H | 12/2020 | NAS2 | 189 | 20 | 33.70 | 18 | 520 | 20 | 26.00 | 4.704 | 19.0 |
| H | 12/2020 | NAT2 | 177 | 20 | 30.64 | 17 | 80 | 20 | 4.00 | 1.799 | 51.0 |
| H | 12/2020 | NAU2 | 163 | 20 | 25.41 | 20 | 360 | 20 | 18.00 | 3.416 | 21.0 |
| N | 11/2020 | NG2 | 289 | 20 | 34.05 | 20 | 380 | 20 | 19.00 | 2.282 | 12.0 |
| N | 11/2020 | NH2 | 275 | 20 | 32.49 | 20 | 840 | 20 | 42.00 | 7.065 | 18.0 |
| N | 11/2020 | NI2 | 192 | 17 | 32.49 | 21 | 400 | 20 | 20.00 | 1.413 | 7.0 |
| T | 12/2019 | NP9 | 219 | 45 | 33.20 | 20 | 780 | 30 | 26.00 | 5.094 | 21.0 |
| T | 12/2019 | NP10 | 267 | 78 | 31.93 | 19 | 570 | 30 | 19.00 | 4.146 | 24.0 |
| T | 12/2019 | NP11 | 192 | 69 | 30.87 | 20 | 690 | 30 | 23.00 | 3.616 | 17.0 |
| W | 12/2020 | WJ2 | 200 | 20 | 29.26 | 19 | 460 | 20 | 23.00 | 3.895 | 18.0 |
| W | 12/2020 | WI2 | 191 | 20 | 30.02 | 20 | 500 | 20 | 25.00 | 4.278 | 18.0 |
| W | 12/2020 | WG2 | 193 | 20 | 29.73 | 20 | 500 | 20 | 25.00 | 5.221 | 23.0 |

**e)** *Pittosporum crassifolium*

| **Site** | **Date** | **Tree** | **Flowers** | | | | **Nectar** | | | | |
| --- | --- | --- | --- | --- | --- | --- | --- | --- | --- | --- | --- |
|  |  |  | **Mean Flower Mass** | | **Mean Flower Size** | | **Total**  **Nectar Volume** | | **Mean Nectar Volume / Flower** | **Mean**  **Sugar / Flower** | **Nectar Concentration** |
|  |  |  |  |  |  |  |  |  |  |  |  |
|  |  |  |  |  |  |  |  |  |  |  |  |
|  | (Month/Year) | ID | (mg) | n | (mm) | n | (µL) | n | (µL) | (mg) | (Brix) |
|  |  |  |  |  |  |  |  |  |  |  |  |
| A | 10/2019 | A1 | 127 | 19 | 8.07 | 13 | 173 | 19 | 9.10 | 0.971 | 11.1 |
| A | 10/2019 | A2 | 68 | 9 | 8.87 | 7 | 38 | 9 | 4.20 | 0.396 | 9.9 |
| A | 10/2019 | A3 | 93 | 8 | 9.20 | 8 | 87 | 8 | 10.90 | 1.359 | 13.1 |
| C | 11/2019 | C14 | 51 | 15 | 7.79 | 13 | 32 | 20 | 1.60 | 0.264 | 17.4 |
| C | 9/2020 | CHJ | 91 | 20 | 9.28 | 20 | 82 | 20 | 4.10 | 0.284 | 7.1 |
| C | 9/2020 | CHK | 83 | 20 | 8.50 | 20 | 46 | 20 | 2.30 | 0.129 | 5.8 |
| C | 9/2020 | CHL | 62 | 20 | 7.79 | 14 | 192 | 20 | 9.60 | 0.133 | 1.4 |
| D | 9/2020 | DO | 99 | 20 | 9.05 | 19 | 270 | 20 | 13.50 | 2.104 | 16.6 |
| D | 9/2020 | DP | 99 | 20 | 9.61 | 16 | 232 | 20 | 11.60 | 2.297 | 21.6 |
| D | 9/2020 | DQ | 108 | 20 | 9.02 | 15 | 318 | 20 | 15.90 | 3.037 | 20.8 |
| H | 10/2020 | NAD | 20 | 20 | 9.13 | 20 | 134 | 20 | 6.70 | 0.739 | 11.5 |
| H | 10/2020 | NAE | 19 | 20 | 9.46 | 20 | 38 | 20 | 1.90 | 0.397 | 22.7 |
| H | 10/2020 | NAF | 19 | 20 | 8.78 | 20 | 130 | 20 | 6.50 | 0.726 | 11.6 |
| N | 9/2020 | NN | 146 | 40 | 9.13 | 20 | 158 | 20 | 7.90 | 0.483 | 6.2 |
| N | 9/2020 | NO | 102 | 25 | 9.46 | 20 | 120 | 20 | 6.00 | 0.578 | 10.0 |
| N | 9/2020 | NP | 106 | 15 | 8.78 | 20 | 172 | 20 | 8.60 | 0.264 | 3.1 |
| T | 09/2019 | NP2 | 112 | 30 | 8.48 | 10 | 168 | 30 | 5.60 | 0.488 | 9.0 |
| T | 09/2019 | NP3 | 90 | 30 | 8.42 | 8 | 68 | 10 | 6.80 | 1.576 | 25.8 |
| W | 9/2020 | WD | 77 | 10 | 9.02 | 10 | 32 | 20 | 1.60 | 0.156 | 10.4 |
| W | 9/2020 | WE | 67 | 40 | 7.57 | 20 | 192 | 20 | 9.60 | 0.808 | 8.7 |
| W | 9/2020 | WF | 83 | 20 | 8.61 | 20 | 200 | 20 | 10.00 | 0.616 | 6.3 |

**f)** *Pittosporum eugenioides*

| **Site** | **Date** | **Tree** | **Flowers** | | | | **Nectar** | | | | |
| --- | --- | --- | --- | --- | --- | --- | --- | --- | --- | --- | --- |
|  |  |  | **Mean Flower Mass** | | **Mean Flower Size** | | **Total**  **Nectar Volume** | | **Mean Nectar Volume / Flower** | **Mean**  **Sugar / Flower** | **Nectar Concentration** |
|  |  |  |  |  |  |  |  |  |  |  |  |
|  |  |  |  |  |  |  |  |  |  |  |  |
|  | (Month/Year) | ID | (mg) | n | (mm) | n | (µL) | n | (µL) | (mg) | (Brix) |
|  |  |  |  |  |  |  |  |  |  |  |  |
| A | 10/2020 | AA | 4 | 20 | 8.26 | 20 | 60 | 20 | 3.00 | 0.346 | 12.3 |
| A | 10/2020 | AB | 12 | 20 | 9.17 | 22 | 48 | 20 | 2.40 | 0.169 | 7.1 |
| C | 9/2020 | CHE | 15 | 40 | 12.32 | 20 | 54 | 20 | 2.70 | 0.402 | 15.7 |
| C | 9/2020 | CHF | 15 | 40 | 13.06 | 20 | 60 | 20 | 3.00 | 0.427 | 15.1 |
| D | 9/2020 | DE | 33 | 20 | 10.62 | 11 | 60 | 20 | 3.00 | 0.100 | 3.4 |
| D | 9/2020 | DF | 26 | 20 | 12.25 | 15 | 62 | 20 | 3.10 | 0.235 | 7.7 |
| D | 9/2020 | DG | 22 | 20 | 10.06 | 13 | 52 | 20 | 2.60 | 0.074 | 2.9 |
| H | 10/2020 | NAR | 13 | 39 | 10.39 | 20 | 46 | 20 | 2.30 | 0.252 | 11.7 |
| H | 10/2020 | NAS | 12 | 40 | 11.12 | 20 | 60 | 20 | 3.00 | 0.658 | 24.6 |
| H | 10/2020 | NAT | 15 | 40 | 12.00 | 20 | 52 | 20 | 2.60 | 0.300 | 12.0 |
| N | 10/2019 | NEL16 | 15 | 20 | 9.71 | 20 | 68 | 20 | 3.40 | 0.228 | 6.9 |
| N | 10/2019 | NEL17 | 18 | 20 | 14.28 | 19 | 58 | 20 | 2.90 | 0.074 | 2.6 |
| N | 10/2019 | NEL18 | 18 | 20 | 12.38 | 20 | 56 | 20 | 2.80 | 0.158 | 5.7 |
| T | 11/2020 | NPG | 14 | 20 | 11.86 | 20 | 78 | 20 | 3.90 | 0.274 | 7.1 |
| T | 11/2020 | NPH | 12 | 20 | 10.16 | 20 | 76 | 20 | 3.80 | 0.109 | 2.9 |
| T | 11/2020 | NPI | 19 | 20 | 10.58 | 20 | 64 | 20 | 3.20 | 0.127 | 4.0 |
| W | 10/2019 | W4 | 20 | 20 | 12.61 | 18 | 122 | 20 | 6.10 | 0.189 | 3.1 |
| W | 10/2019 | W5 | 13 | 15 | 9.91 | 15 | 89 | 15 | 5.90 | 0.133 | 2.3 |
| W | 10/2019 | W6 | 16 | 15 | 12.18 | 15 | 93 | 15 | 6.20 | 0.158 | 2.6 |

**g)** *Pittosporum tenuifolium*

| **Site** | **Date** | **Tree** | **Flowers** | | | | **Nectar** | | | | |
| --- | --- | --- | --- | --- | --- | --- | --- | --- | --- | --- | --- |
|  |  |  | **Mean Flower Mass** | | **Mean Flower Size** | | **Total**  **Nectar Volume** | | **Mean Nectar Volume / Flower** | **Mean**  **Sugar / Flower** | **Nectar Concentration** |
|  |  |  |  |  |  |  |  |  |  |  |  |
|  |  |  |  |  |  |  |  |  |  |  |  |
|  | (Month/Year) | ID | (mg) | n | (mm) | n | (µL) | n | (µL) | (mg) | (Brix) |
|  |  |  |  |  |  |  |  |  |  |  |  |
| A | 10/2019 | A9 | 67 | 17 | 6.51 | 10 | 60 | 10 | 6.00 | 0.158 | 2.7 |
| A | 10/2019 | A11 | 84 | 10 | 7.55 | 10 | 54 | 10 | 5.40 | 0.024 | 0.4 |
| A | 10/2019 | A20 | 87 | 15 | 6.69 | 10 | 51 | 10 | 5.10 | 0.106 | 2.1 |
| C | 11/2020 | C13 | 46 | 15 | 7.21 | 15 | 83 | 15 | 5.50 | 0.087 | 1.6 |
| C | 11/2020 | C15 | 51 | 15 | 8.68 | 14 | 56 | 15 | 3.70 | 0.183 | 5.0 |
| C | 11/2020 | C16 | 55 | 30 | 8.18 | 28 | 53 | 15 | 3.50 | 0.112 | 3.3 |
| D | 9/2020 | DR | 69 | 20 | 8.92 | 16 | 118 | 20 | 5.90 | 0.117 | 2.0 |
| D | 9/2020 | DT | 75 | 20 | 4.38 | 20 | 78 | 20 | 3.90 | 0.268 | 7.0 |
| H | 10/2020 | NAA | 50 | 40 | 6.77 | 20 | 18 | 20 | 0.90 | 0.170 | 20.5 |
| H | 10/2020 | NAB | 44 | 40 | 7.15 | 19 | 46 | 20 | 2.30 | 0.320 | 14.5 |
| H | 10/2020 | NAC | 40 | 35 | 6.57 | 20 | 50 | 20 | 2.50 | 0.383 | 16.5 |
| N | 10/2019 | NEL9 | 87 | 11 | 7.90 | 11 | 44 | 11 | 4.00 | 0.961 | 26.5 |
| N | 10/2019 | NEL10 | 53 | 10 | 7.55 | 10 | 36 | 10 | 3.60 | 0.176 | 5.0 |
| N | 10/2019 | NEL11 | 74 | 12 | 7.86 | 12 | 62 | 12 | 5.20 | 0.161 | 3.1 |
| T | 11/2020 | NPK | 97 | 20 | NA | NA | 60 | 20 | 3.00 | 0.570 | 20.5 |
| W | 10/2019 | W1 | 69 | 10 | 6.42 | 10 | 29 | 10 | 2.90 | 0.159 | 5.5 |
| W | 10/2019 | W2 | 64 | 10 | 7.03 | 10 | 32 | 10 | 3.20 | 0.174 | 5.5 |
| W | 10/2019 | W3 | 67 | 10 | 7.28 | 10 | 34 | 10 | 3.40 | 0.434 | 13.5 |

**h)** *Sophora microphylla*

| **Site** | **Date** | **Tree** | **Flowers** | | | | **Nectar** | | | | |
| --- | --- | --- | --- | --- | --- | --- | --- | --- | --- | --- | --- |
|  |  |  | **Mean Flower Mass** | | **Mean Flower Size** | | **Total**  **Nectar Volume** | | **Mean Nectar Volume / Flower** | **Mean**  **Sugar / Flower** | **Nectar Concentration** |
|  |  |  |  |  |  |  |  |  |  |  |  |
|  |  |  |  |  |  |  |  |  |  |  |  |
|  | (Month/Year) | ID | (mg) | n | (mm) | n | (µL) | n | (µL) | (mg) | (Brix) |
|  |  |  |  |  |  |  |  |  |  |  |  |
| A | 10/2019 | A6 | 1105 | 19 | 49.98 | 11 | 1064 | 19 | 56.00 | 6.264 | 11.7 |
| A | 10/2019 | A7 | 666 | 10 | 51.72 | 10 | 120 | 10 | 12.00 | 0.660 | 5.5 |
| A | 10/2019 | A8 | 827 | 20 | 52.65 | 10 | 680 | 20 | 34.00 | 3.443 | 10.4 |
| C | 9/2020 | CHB | 895 | 40 | 43.26 | 20 | 600 | 20 | 30.00 | 10.753 | 42.5 |
| C | 9/2020 | CHC | 947 | 40 | 40.26 | 20 | 500 | 20 | 25.00 | 6.794 | 30.4 |
| C | 9/2020 | CHD | 338 | 30 | 31.24 | 20 | 100 | 20 | 5.00 | 2.332 | 53.0 |
| D | 9/2020 | DU | 465 | 40 | 30.94 | 10 | 440 | 20 | 22.00 | 5.352 | 27.1 |
| D | 9/2020 | DV | 837 | 40 | 42.50 | 19 | 1160 | 20 | 58.00 | 10.961 | 20.7 |
| D | 9/2020 | DW | 418 | 40 | 36.18 | 17 | 440 | 20 | 22.00 | 4.585 | 23.1 |
| H | 10/2019 | N1 | 821 | 20 | 49.06 | 20 | 1440 | 20 | 72.00 | 9.484 | 14.0 |
| H | 10/2020 | N2 | 827 | 20 | 47.31 | 20 | 860 | 20 | 43.00 | 6.092 | 15.2 |
| H | 10/2020 | N3 | 737 | 17 | 50.01 | 17 | 816 | 17 | 48.00 | 6.263 | 13.7 |
| N | 10/2019 | NEL12 | 783 | 15 | 53.04 | 15 | 570 | 15 | 38.00 | 12.912 | 40.1 |
| N | 10/2019 | NEL26 | 741 | 20 | 53.76 | 20 | 450 | 15 | 30.00 | 4.118 | 14.5 |
| N | 10/2019 | NEL27 | 652 | 20 | 49.35 | 20 | 840 | 15 | 56.00 | 9.315 | 17.8 |
| N | 10/2019 | NEL28 | 546 | 20 | 50.69 | 20 | 780 | 15 | 52.00 | 8.120 | 16.8 |
| T | 09/2019 | NP1 | 608 | 60 | 42.99 | 60 | 270 | 30 | 9.00 | 3.131 | 41.9 |
| T | 09/2019 | NP4 | 1116 | 30 | 49.93 | 30 | 1050 | 30 | 35.00 | 8.930 | 28.4 |
| W | 10/2019 | W8 | 1076 | 14 | 47.12 | 15 | 420 | 15 | 28.00 | 7.671 | 31.7 |
| W | 10/2019 | W9 | 830 | 16 | 47.68 | 15 | 375 | 15 | 25.00 | 6.777 | 30.1 |
| W | 10/2019 | W10 | 632 | 14 | 43.76 | 15 | 360 | 15 | 24.00 | 7.270 | 34.1 |

**Table S2a-c:** Overview of species’ site mean trait minima (‘min’) and maxima (’max’) values, with **a)** percentage of x out of all spp per site, **b)** percentage per climate zone, **c)** percentage per sampled New Zealand main island. Highest percentages of minima and maxima per trait across all species are marked in bold.

**a)** (by site; values for each site and trait in % of total minima or maxima across all species)

| **Site** | | | **Nectar** | | | | | | | | | **Flowers** | | | | | |
| --- | --- | --- | --- | --- | --- | --- | --- | --- | --- | --- | --- | --- | --- | --- | --- | --- | --- |
|  |  |  | **Volume** | | | **Sugar** | | | **Concentration** | | | **Mass** | | | **Size** | | |
|  |  |  |  |  |  |  |  |  |  |  |  |  |  |  |  |  |  |
|  |  |  | **min** | **-** | **max** | **min** | **-** | **max** | **min** | **-** | **max** | **min** | **-** | **max** | **min** | **-** | **max** |
| **A** | | | 25 |  | 13 | **50** |  | 0 | **38** |  | 0 | 13 |  | 13 | 13 |  | 0 |
| **T** | | | 0 |  | 0 | 13 |  | 13 | 0 |  | 13 | 13 |  | 13 | 13 |  | 0 |
| **W** | | | 0 |  | 13 | 0 |  | 13 | 13 |  | 13 | 0 |  | 25 | 13 |  | 0 |
| **H** | | | 25 |  | 25 | 13 |  | 25 | 13 |  | 25 | **50** |  | 13 | 13 |  | 0 |
| **N** | | | 13 |  | 0 | 0 |  | 0 | 25 |  | 0 | 0 |  | 13 | 13 |  | 38 |
| **C** | | | **38** |  | 0 | 13 |  | 13 | 0 |  | 25 | 13 |  | 0 | 13 |  | 25 |
| **D** | | | 0 |  | **50** | 25 |  | **38** | 13 |  | 25 | 13 |  | **38** | 25 |  | 38 |

**b)** (by climate zone; values for each zone and trait in % of total minima or maxima across all species)

| **Climate**  **Zone** | **Nectar** | | | | | | | | | **Flowers** | | | | | |
| --- | --- | --- | --- | --- | --- | --- | --- | --- | --- | --- | --- | --- | --- | --- | --- |
|  | **Volume** | | | **Sugar** | | | **Concentration** | | | **Mass** | | | **Size** | | |
|  |  |  |  |  |  |  |  |  |  |  |  |  |  |  |  |
|  | **min** | **-** | **max** | **min** | **-** | **max** | **min** | **-** | **max** | **min** | **-** | **max** | **min** | **-** | **max** |
| **N-NI** | 25 |  | 13 | **50** |  | 0 | **38** |  | 0 | 13 |  | 13 | 13 |  | 0 |
| **SW-NI** | 0 |  | 13 | 13 |  | 25 | 13 |  | 25 | 13 |  | **38** | 25 |  | 0 |
| **E-NI** | 25 |  | 25 | 13 |  | 25 | 13 |  | 25 | **50** |  | 13 | 13 |  | 0 |
| **N-SI** | 13 |  | 0 | 0 |  | 0 | 25 |  | 0 | 0 |  | 13 | 13 |  | **38** |
| **E-SI** | **38** |  | **50** | 38 |  | **50** | 13 |  | **50** | 25 |  | **38** | **38** |  | **63** |

**c)** (by island; values for each island and trait in % of total minima or maxima across all species)

| **Island** | **Nectar** | | | | | | | | | | **Flowers** | | | | | |
| --- | --- | --- | --- | --- | --- | --- | --- | --- | --- | --- | --- | --- | --- | --- | --- | --- |
|  | **Volume** | | | | **Sugar** | | | **Concentration** | | | **Mass** | | | **Size** | | |
|  |  |  |  |  |  |  |  |  |  |  |  |  |  |  |  |  |
|  | **min** | | **-** | **max** | **min** | **-** | **max** | **min** | **-** | **max** | **min** | **-** | **max** | **min** | **-** | **max** |
| **North** |  | 50 |  | 50 | **75** |  | 50 | **63** |  | 50 | **75** |  | 50 | **75** |  | 0 |
| **South** |  | 50 |  | 50 | 38 |  | 50 | 38 |  | 50 | 25 |  | 50 | 50 |  | **100** |

**Table S3:**  Statistical parameters of GAMM analysis on regional variation in plant traits and their correlations with tested environmental factors.

| **Spp** | **n** | **Nectar** | | | **Flower** | |
| --- | --- | --- | --- | --- | --- | --- |
|  |  | **Volume** | **Sugar** | **Concentration** | **Mass** | **Size** |
|  |  |  |  |  |  |  |
| **CA** | 18 | - | R² = 0.514,  *P* = 0.017 (MAR),  *P* = 0.006 (MAT),  Tweedie(2), log | R² = 0.580,  *P* < 0.001 (MAT),  *P* < 0.001 (MRH),  Tweedie(2), log | - | - |
| **FE** | 20 | - | - | - | n.s. | R² = 0.437,  *P* = 0.027 (MSH),  Tweedie(2), log |
| **LS** | 20 | R² = 0.643,  *P* < 0.001 (MSH),  Tweedie(2), log | - | - | - | n.s. |
| **ME** | 20 | - | - | R² = 0.372,  *P* = 0.047 (MAR),  Tweedie(1.8), log | R² = 0.847,  *P* = 0.02 (MRH),  *P* < 0.001 (MSH),  Tweedie(2), log, n=18 | R² = 0.559,  *P* = 0.001 (MSH),  Tweedie(2), log |
| **PC** | 21 | R² = 0.424,  *P* = 0.04 (MSH),  Tweedie(1.8), log | R² = 0.799,  *P* = 0.019 (MSH),  *P* = 0.003 (DBH),  Tweedie(2), log | - | R² = 0.641,  *P* < 0.001 (MRH),  Tweedie(2), log | - |
| **PE** | 19 | R² = 0.183,  P = 0.05(MRH),  Tweedie(2), log | - | R² = 0.324,  *P* = 0.017(MRH),  Tweedie(2), log | R² = 0.538,  *P* < 0.001(MAT),  Tweedie(2), log | - |
| **PT** | 18 | R² = 0.437,  *P* = 0.007(MRH),  Gamma, inverse | - | - | R² = 0.596,  *P* = 0.005(MRH),  *P* = 0.003(MAR),  Gamma, inverse | - |
| **SM** | 21 | - | - | R² = 0.297,  *P* = 0.008 (MAT),  Gamma, inverse | - | R² = 0.626,  *P* = 0.001 (MAT),  *P* = 0.002 (MSH),  Gamma, inverse |
|  | |  |  |  |  |  |
| **R² - range** | | **0.2 - 0.6** | **0.5 - 0.8** | **0.3 - 0.6** | **0.5 - 0.8** | **0.4 - 0.6** |
|  | |  |  |  |  |  |
| **MAR** | | **X** | **X** | **X** | **X** |  |
| **MAT** | |  | **X** | **X** | **X** | **X** |
| **MRH** | | **X** |  |  | **X** |  |
| **MSH** | | **X** | **X** |  | **X** | **X** |
| **DBH** | |  | **X** |  |  |  |
